# microRNA profile of *Hermetia illucens* (black soldier fly) and its implications on mass rearing

**DOI:** 10.1371/journal.pone.0265492

**Published:** 2022-03-17

**Authors:** Sarah DeRaedt, Anandi Bierman, Peter van Heusden, Cameron Richards, Alan Christoffels

**Affiliations:** 1 South African National Bioinformatics Institute, South African Medical Research Council Bioinformatics Unit, The University of the Western Cape, Bellville, Western Cape, South Africa; 2 AgriProtein Technologies (Pty) Limited, Philippi, Western Cape, South Africa; Universita degli Studi della Basilicata, ITALY

## Abstract

The growing demands on protein producers and the dwindling available resources have made *Hermetia illucens* (the black soldier fly, BSF) an economically important species. Insights into the genome of this insect will better allow for robust breeding protocols, and more efficient production to be used as a replacement of animal feed protein. The use of microRNA as a method to understand how gene regulation allows insect species to adapt to changes in their environment, has been established in multiple species. The baseline and life stage expression levels established in this study, allow for insight into the development and sex-linked microRNA regulation in BSF. To accomplish this, microRNA was extracted and sequenced from 15 different libraries with each life stage in triplicate. Of the total 192 microRNAs found, 168 were orthologous to known arthropod microRNAs and 24 microRNAs were unique to BSF. Twenty-six of the 168 microRNAs conserved across arthropods had a statistically significant (p < 0.05) differential expression between Egg to Larval stages. The development from larva to pupa was characterized by 16 statistically significant differentially expressed microRNA. Seven and 9 microRNA were detected as statistically significant between pupa to adult female and pupa to adult male, respectively. All life stages had a nearly equal split between up and down regulated microRNAs. Ten of the unique 24 miRNA were detected exclusively in one life stage. The egg life stage expressed five microRNA (*hil-miR-m*, *hil-miR-p*, *hil-miR-r*, *hil-miR-s*, and *hil-miR-u)* not seen in any other life stages. The female adult and pupa life stages expressed one miRNA each *hil-miR-h* and *hil-miR-ac* respectively. Both male and female adult life stages expressed *hil-miR-a*, *hil-miR-b*, and *hil-miR-y*. There were no unique microRNAs found only in the larva stage. Twenty-two microRNAs with 56 experimentally validated target genes in the closely related *Drosophila melanogaster* were identified. Thus, the microRNA found display the unique evolution of BSF, along with the life stages and potential genes to target for robust mass rearing. Understanding of the microRNA expression in BSF will further their use in the crucial search for alternative and sustainable protein sources.

## Introduction

The world population is projected to reach 9.8 billion by the year 2050 [1], meaning food production needs to increase by 50% [2]. However, food production is expected to decrease by as much as 25% by 2050 due to climate change and unsustainable traditional methods of manufacturing food [2]. To combat this decline, new methods of attaining the three major resources involved in food production including food, water, and energy, must be found [3]. Due to the increasing demands for protein, it is important to explore sustainable, alternative, large-scale protein sources which are environmentally friendly.

Insects are one alternative source of protein, with an estimated 2,000 species already being consumed mainly in low- and middle-income countries [4, 5]. The black soldier fly (*Hermetia illucens;* BSF) is a species found worldwide and can be used as a sustainable animal and fish feed [6–9]. Depending on the organic substrate the black soldier fly larvae (BSFL) are fed and the extraction process, they can contain a range of 40–60% protein, up to 47% lipid, and 3–8% chitin in prepupae [10–15]. This makes them not only a good protein source, but shows potential for a variety of derived products, and has minimal environmental impacts. Due to these facts, and that they are not a nuisance species, or mechanical disease vector [16, 17], they are an ideal candidate for large scale rearing for food sustainability and waste reduction [18, 19].

The potential uses for BSF are numerous; however, until recently little has been done to fully understand their genetics and the implications of mass rearing. Many studies have explored the midgut microbiota [20–23]. In 2015, a draft genome was published [24], and in 2020 the full genome was sequenced at a depth of 300x coverage [25]. Another study still in preprint, has covered the genome using long read PacBio sequencing [26]. The midgut transcriptome, and the application of the gene modification tool CRISPR/Cas-9 have also been recently reported for adapting BSF to industrialization [25]. Multiple insect genomics studies barring BSF have demonstrated the role of microRNA in regulating biological and behavioural functions at different life stages [27–30]. As arthropod genetic diversity is known to diminish through colonization [17, 31], looking directly at gene regulation via microRNA expression explores how the BSF adapts to the mass rearing environment. Mapping the baseline microRNA expression data will provide crucial information on the large-scale development of BSF into an economically sustainable protein source. This study creates a database of novel microRNAs and their expression levels across five life stages and two sexes in BSF and provides candidate microRNAs with potential impact on BSF mass rearing.

## Results

### Sequencing and mapping

The microRNA from five different life stages, (egg, larva, pupa, adult unmated female, and adult unmated male) of *H*. *illucens* were extracted and sequenced to complete a baseline assessment of the species. Illumina TruSeq Small Library platform was used to create the microRNA libraries. The total raw read count for the life stages ranged from 10.6–16.4 million reads with an average sequence length of 51 nucleotides (Table 1). More than 98% of the reads had Phred quality values (PQV) of 20 and 96% with PQV of 30. After the reads were filtered for lengths smaller than 18 nucleotides and any non-canonical values, the average reads were 10.9, 9.1, 11.4, 12.1, and 10.5 million for egg, larva, pupa, adult unmated female, and adult unmated male, respectively.

**Table 1:** Summary of BSF reads sequenced and mapped.

Samples	Total Raw Reads	Total Filtered Reads	Mapped Reads	Unmapped Reads	Mapped (%)	Unmapped (%)
1E	10634038	10485727	202996	10282731	1.9%	98.1%
2E	11391345	11058752	312488	10746264	2.8%	97.2%
3E	11630268	11143061	471985	10671076	4.2%	95.8%
E avg	11218550	10895847	329156	10566690	3.0%	97.0%
1L	13716268	11013749	46243	10967506	0.4%	99.6%
**2L**	**16363975**	**5374681**	**18318**	**5356363**	**0.3%**	**99.7%**
3L	14753374	1077735	74337	10703020	0.7%	99.3%
L avg	14944539	5822055	46299	9008963	0.5%	99.5%
1P	11717837	10156757	157402	9999355	1.5%	98.5%
2P	14142734	11927696	265560	11662136	2.2%	97.8%
3P	12674729	11982861	89089	11893772	0.7%	99.3%
P avg	12845100	11355771	170684	11185088	1.5%	98.5%
1M	10958506	9495353	955239	8540114	10.1%	89.9%
2M	12428650	11973265	474068	11499197	4.0%	96.0%
3M	10665444	10037438	527529	9509909	5.3%	94.7%
M avg	11350867	10502019	652279	9849740	6.5%	93.5%
1F	13126901	12795661	585428	12210233	4.6%	95.4%
2F	14284210	13860109	579082	13281027	4.2%	95.8%
3F	11691808	9695009	1560275	8134734	16.1%	83.9%
F avg	13034306	12116926	908262	11208665	8.3%	91.7%

Abbreviations: E = egg; L = larva; P = pupae; F = female; M = male; 1–3 = replicate; avg = average of 3 replicates.

The percentages of total filtered reads that mapped to the BSF genome were: 3.0% of egg, 0.5% larva, 1.4% pupa, 8.2% female, and 6.4% male libraries. The second larval replicate only had 5.4 million reads after filtering which lowered the overall larval library mapping (Table 1).

### Identification of novel microRNAs in *Hermetia illucens*

Analysis of the 15 BSF libraries was completed using MiRDeep2 software (v.2.0.1.2), and alignment of all known arthropod microRNAs from miRbase v22.1 was used to discover baseline microRNAs of BSF. As BSF is a novel species with no known/reported microRNA, all microRNA found were considered novel. Any microRNA identified to be conserved across other known arthropods are referred to as conserved, and the microRNA that have yet to be identified in any other species are defined as unique microRNA. In order to be considered unique, both the mature and star sequence needed to be present and found in at least 2 of the 15 libraries. A total of 192 novel microRNAs were found across the 15 BSF libraries. Of these 168 were found to be orthologous to known arthropod microRNAs (S1 Table), and 24 were found to be unique microRNA of BSF (S2 Table), not identified in any other species.

### Differential expression

microRNA differential expression was calculated with log fold changes (LFC) of 0, 2, 5, and 10 in expression between life stages of egg to larva, larva to pupa, pupa to adult female, pupa to adult male. Twenty-six of the 168 microRNAs conserved across arthropods had a statistically significant (p-adjusted value < 0.05) differential expression between Egg to Larval stages. Nearly half (14) of these 26 microRNA were downregulated and 12 were upregulated. Development from Larva to Pupa was characterized by 16 statistically significant (p-adjusted value < 0.05) differentially expressed microRNA with equal amount up and downregulated transcripts. Differential expression of seven miRNA were detected as statistically significant (p-adjusted value < 0.05) between Pupa to Adult Female (3 miRNA up regulated and 4 microRNAs down regulated). microRNAs from Pupa to Adult Male had 9 statistically significant expression changes (4 miRNA up regulated and 5 down regulated (Table 2)).

**Table 2:** microRNA with statistically significant regulation between life stages of novel BSF microRNA with the same seed as known arthropod microRNA.

Life Stage	Egg to Larva	Larva to Pupa	Pupa to Female	Pupa to Male
Up Regulated	12	8	3	4
Down Regulated	14	8	4	5
Total miRNA w/padj<0.05	26	16	7	9
Total miRNA	168	168	168	168

**Fig 1:**
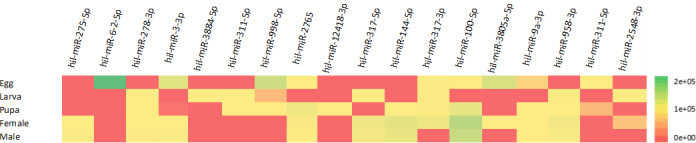
Heatmap of conserved microRNA expression with a log fold change (LFC) of ±10 (p-adjusted value <0.05) across life stages.

A total of 44 miRNAs (23% of the total microRNAs) showed differential expression between at least two stages with an LFC of ±2. These included 31 miRNAs conserved across all arthropods and 13 unique to BSF. A total of 40 miRNAs (21% of the total microRNAs) showed differential expression between at least two life stages with an LFC of ±5 (30 conserved across all arthropods and 10 unique to BSF). Finally, 21 microRNA were identified with an LFC of ±10 (11% of total miRNA). These included 18 miRNAs conserved across arthropods and 3 miRNAs unique to BSF (Fig 1; Table 3).

**Table 3:** Summary of microRNAs differential expression log fold change (LFC).

Log Fold Change	Total miRNA	LFC ± 2	LFC ± 5	LFC ± 10
**Known arthropod miRNA**	168	31	30	18
***H*. *illucens* unique**	24	13	10	3
**Total miRNA**	192	44	40	21

**Fig 2:**
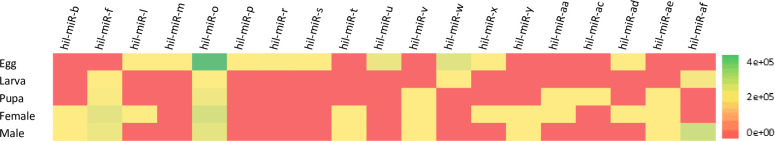
Heatmap of unique microRNA expression across life stages. Expression is based on average read count of triplicate samples for each life stage.

Seven of the unique 24 miRNA were detected exclusively in one life stage (Fig 2). The egg life stage expressed five miRNAs not seen in any other life stages (*hil-miR-m*, *hil-miR-p*, *hil-miR-r*, *hil-miR-s*, and *hil-miR-u*; S2 Table). The female adult and pupa life stages expressed one miRNA each (*hil-miR-h* and *hil-miR-ac* respectively; S2 Table). Both male and female adult life stages expressed 3 shared miRNA (*hil-miR-a*, *hil-miR-b*, and *hil-miR-y;* S2 Table). There were no unique microRNAs found only in the larva stage (Fig 2).

### Predicted target genes

BSF miRNAs were searched against experimentally validated target genes in *D*. *melanogaster* using Flybase.org (FB2020_06) [32] and MirTarBase (http://mirtarbase.cuhk.edu.cn/php/index.php) [33] to extrapolate BSF genes targeted for miRNA regulation. *D*. *melanogaster* was chosen as the closest relative to BSF with experimentally validated target genes. These databases represent curated published, and experimentally validated microRNA-Target Gene pathways. Fifty-six genes were found to be targets of 22 BSF microRNAs. The target genes covered a range of developmental functions in Drosophila (Table 4). In the absence of experimentally verified BSF miRNA targets, the closely related insect species (*D*. *melanogaster)*, provided greater understanding of the development of the fly during rearing. The predicted target genes are characterized by 21 known gene families, the majority identified as C2H2 Zinc Finger Transcription Factors gene family. The second highest representative family were the Basic Helix-Loop-Helix Transcription Factors (negative regulators of notch signaling pathway), followed by RHG Proteins family. The Bearded Gene Family and ABCG ATP-Binding Cassette Transporter Subfamily matched two genes each. The other 16 gene families were each represented by single genes. Each of these genes were targeted by multiple microRNAs.

**Table 4:** Novel *H*. *illucens* microRNA with orthologous microRNA and their predicted target genes in *Drosophila*.

Novel *H*. *illucens* miRNA with same seed	Known Predicted Target Gene in *Drosophila*	Egg to Larva	Larva to Pupa	Pupa to Female	Pupa to Male
		LFC*	LFC*	LFC*	LFC*
hil-bantam-3p	W	2.563	-0.926	-0.78	-1.172
hil-let-7-5p	ab	2.074	8.84	0.832	0.192
hil-miR-124-3p	ana, gli	-4.198	5.269	1.485	0.65
hil-miR-1-3p	Amyotrophic lateral sclerosis 2, CG11377, CG17065, CG18542, CG31121, Chd64, crim, DI, Jafrac2, Msr-110, Nedd4, sinu, tutl	0.403	0.48	-1.166	-1.493
hil-miR-14-3p	EcR, Ice, IP3K2, sug	1.84	0.078	-0.675	-0.828
hil-miR-263a-5p	W	-0.335	2.089	1.466	1.184
hil-miR-276a-3p	DopR	1.816	2.127	0.417	0.008
hil-miR-278-3p	ex, tup	10.304	-0.466	2.175	1.362
hil-miR-279-3p	esg, nerfin-1, os, SP555	-3.171	0.839	0.388	0.604
hil-miR-283-5p	cos, smo	1.562	-2.857	-0.412	-0.267
hil-miR-2a-3p	grim, reaper, skl, hid, malpha, HLHmdelta	-3.466	1.563	0.821	0.693
hil-miR-315-5p	Axn, Notum	-1.824	1.066	0.843	0.772
hil-miR-316-5p	IA-2	7.816	0.174	2.418	1.813
hil-miR-317-3p	yellow-c	1.164	-1.118	1.486	14.041
hil-miR-34-5p	Eip74EF, Su(z)12	6.163	-6.769	-6.126	-5.636
hil-miR-5-5p	smo	2.074	0	-4.82	-6.715
hil-miR-7-5p	e, fng, h, HLHm5, I(1)MZ4, iHog, ttk	-9.097	3.818	-0.892	0.181
hil-miR-8-3p	CG13060, CG32767, CG8420, Cpr56F, ena, Gug, pan, ush, wls	2.353	-0.304	-0.043	0.173
hil-miR-92a-3p	sha	-7.694	2.342	0.993	2.052
hil-miR-9a-5p	Bx, dg, sens	-2.725	-0.306	1.621	1.746
hil-miR-iab-4-5p	abd-A, Ubx	-6.179	4.019	-0.978	1.564
hil-miR-iab-8-5p	abd-A, Abd-B, Ubx	2.074	0	0	-7.464

*LFC = Log Fold Change between life stages listed.

## Discussion

BSF has become an insect of great economic and environmental value over the past decades. This study established a baseline survey of novel microRNAs, both conserved and unique, in the BSF and their expression levels across 5 different life stages and identified likely target genes for these miRNAs. Like most species, the BSF genome retains a highly conserved microRNA library as evidenced by the large numbers of orthologous microRNAs found from a wide range of arthropod species in the miRBase database. Focusing our miRNA discovery pipeline on known arthropod species miRNA only, allowed for a stronger comparative analysis of potential microRNA function in BSF. Finding orthologs of microRNAs across species results in a higher power of discovery for *de novo* microRNAs [34]. The high number of orthologs found for each microRNA family, along with the strict identification criterion utilized by the miRDeep2 program for mature, star, and precursor sequences, provide high confidence in the microRNAs found in the BSF genome. These new BSF microRNA add another species that follow the highly conserved nature of microRNA across all species.

Overall, 192 novel microRNAs were found in the black soldier fly, with the vast majority conserved (87%). The 24 unique microRNAs that passed the criteria to be included, came from a group of 91 potentially unique sequences found. The criteria used ensured that reporting would only include highly likely candidates. However, the many sequences not considered candidates, represent a potential larger network of microRNAs for future study. The limited number of libraries per life stage (3) could mean some candidate sequences were missed. BSF has already been seen to have a divergent and large genomic landscape [25], and the number of microRNAs follows suit.

### Stage specific miRNA expression

Understanding the basic expression levels across the life stages has led to a better understanding of stage specific microRNA regulation. Seventy-four conserved microRNAs were found to be specific to a single life stage, while only 27 were found across all life stages (S1 Table). These may be important for gene regulation linked to life stage specific development. The unique microRNAs identified had a number of life stage specific microRNAs: (Egg) *hil-miR-m*, *hil-miR-p*, *hil-miR-r*, *hil-miR-s*, *hil-miR-u*, (Pupa) *hil-miR-ac*, (Female) *hil-miR-h*, (Male & Female) *hil-miR-a*, *hil-miR-b*, and *hil-miR-y*. These 10 represent nearly half (42%) of the unique microRNAs found. This life stage specificity may be due to too low frequencies of the fragments to be detected through sequencing in the other life stages. However, these 10 unique microRNAs provide insight into the egg, larva, pupa, and adult life stages of the BSF and can be used to compare the developmental regulatory differences of microRNAs to those of other insect species [35]. The inclusion of separate male and female life stages allows insight into the sex-linked microRNAs of BSF, and their potential functions in development in sex differentiation [36, 37]. For instance, *hil-miR-h*, *hil-miR-317-3p*, *hil-miR-3805a-5p*, and *hil-miR-2548-3p* displayed different expression levels between female and male samples. The unique microRNA of *hil-miR-af* was found in the larval and male stages, but not the female life stage. These stage specific microRNAs provide valuable developmental information unique to BSF showing their genetic adaptability and divergence from other insect species [25, 38, 39].

Eighteen conserved microRNAs were found with substantial (LFC 10) expression regulation between at least two life stages. While these do not have any known experimentally validated predicted target genes, the level of differential expression makes them microRNA of interest for future study.

### miRNA target prediction

Looking at the functions of possible target genes highlights how important the regulation of microRNA is in the development of insects. While most of the predicted target genes deal with cell development, differentiation, and death; the target genes discussed below were selected for having the greatest potential impacts on the mass rearing of BSF.

*bantam-3p* has been well established in *D*. *melanogaster* and involved in the cell differentiation, apoptosis, neural development, and germ line maintenance [40, 41]. The microRNA is expressed during the 3^rd^ larval instar stage, regulating optical disc and photo receptor differentiation [41]. b*antam* is linked to mediating the circadian rhythm proteins (*clk*) in *D*. *melanogaster*. Overexpression of *bantam* causes a lengthening of the circadian period [42]. BSF *bantam* expression follows a path consistent with the developmental role it has been identified to play with higher expression in larva, followed by a decreasing trend from larva to pupa, and both adult stages where it was heavily downregulated. Both factors may be exploited for optimal mass rearing as BSF have been shown to have a direct relationship between length of light exposure and egg production [43].

The *let-7* microRNA is one of the first to be identified in *C*. *elegans* and is known to be highly conserved across most species [44]. Controlling expression of *let-7* is essential to prevent deleterious phenotypes such as wing, fertility, motility, and flight deficiencies from unusual abdominal musculature maturation [44–48]. *let-*7 has been shown to regulate the ecdysis pathways during molting stages in ticks [49] and silkworms [50]. The microRNA has been seen to increase in expression over the third larval instar, with highest expression levels in the pupa stage, in *D*. *melanogaster* [44]. BSF *let-*7 follows the same expression pattern, peaking in expression during the pupal stage.

Overexpression of *mir-14* leads to lean *D*. *melanogaster* [51]. *Sugarbabe* is one of the target genes for *mir-14* and has been shown to be controlled by both diet nutrient levels and *mir-14* targeting in the face of nutrient deprivation [52]. The non-nutrient dependent nature of *mir-14* regulation has led to flies being obese during starvation [52]. As BSFL fat content is a known beneficial part of the role of BSF as sustainable feed additive [53], being able to potentially increase fat content would be a useful tool in the mass rearing process.

### Future work

The baseline of conserved and unique novel microRNAs in the BSF genome were all identified by utilizing established computational methods. As BSF is a novel species with a relatively new genome, the baseline data identified here should be confirmed through laboratory validation. These microRNA sequences should be used to confirm the predicted target genes and regulatory pathways. Once validated, the microRNA can be harnessed in breeding protocols to improve mass rearing of BSF by understanding how they adapt to different environments [28, 46, 54] and is being used in the grape and olive industries [55, 56].

### Conclusions

The conserved and unique microRNA described in this study form an essential understanding of gene regulation in the economically and environmentally important species of *H*. *illucens* and will provide potential targets for genetic manipulation of this species in order to improve its use as an alternative protein source.

## Methods

### Sample collection

*Hermetia illucens* (black soldier fly, BSF) were reared under factory breeding conditions by AgriProtein Technologies Ltd in Philippi, South Africa. The flies were fed *ad libitum* on a standardized proprietary diet based on the commercial composition of layer hen feed. All life stages were kept at 28°C (±2°C), 80% relative humidity, under 12-hour day and night cycles.

Fifteen specimens were selected across five life stages: egg, larva, pupa, adult unmated female, and adult unmated male. The 15 specimens were triplicates for each of the five life stages. In order to acquire enough material to extract microRNAs according to methodology, an egg batch laid from a single female was used for each egg replicate. The egg batches were laid one day prior to collection. Larvae were collected during the L5 instar, which was defined as day 21 (±2) from egg. The L5 instar was selected due to its size and ease of identification, along with the life stage’s importance in protein production. The pupal stage was defined as day 28 (±2) from egg. Adult females and males were collected before being allowed to mate.

### microRNA extraction

All specimens were harvested on the same day and transported to the lab in sterile falcon tubes. The flies were left to acclimate to the lab for 60 minutes after transport to allow any stress from travel to reduce. The samples were then flash frozen with liquid nitrogen and ground into a powder. The lysing agent QIAzol was used, and the microRNA extraction kit miRNeasy Mini Kit from Qiagen [57] standard protocols were followed. A Qbit RNA Assay (Invitrogen™) was performed for quality control and to ensure enough microRNA was extracted for sequencing. After extraction, all samples were stored at -80°C. To ship samples for sequencing, the microRNA was stabilized at room temperature using RNAstable^®^ (Biomatrica^®^).

### Sequencing

Extracted samples were shipped to Macrogen Inc (Seoul, South Korea) for small RNA sequencing. Illumina TruSeq Small RNA Library construction of the 15 samples was completed. Sequencing was done on an Illumina HiSeq 2500 machine with 8 million reads per sample.

### Read processing and analysis

Sequences from the 15 different libraries were quality checked using FastQC (http://www.bioinformatics.babraham.ac.uk/projects/fastqc/) [58]. Reads were filtered for any non-canonical letters, and the 3’ adapters were trimmed, followed by the removal of any reads shorter than 18 nucleotides long using the MiRDeep2 (v.2.0.1.2) program [59]. The genome of *Hermetia illucens* was used to map the reads (NCBI: assembly iHerIll2.2.curated20191125). The genome was indexed for mapping using Bowtie (v1.1.1) [60].

After mapping the reads, MiRDeep2 was used to identify any exact matches to published microRNAs (miRBase, v22.1) [61–68] found in the sample reads. Initially, all known microRNAs from miRBase were used, then filtered for arthropod species only for more closely related relevance of the microRNAs. The microRNAs found were given read counts using the Quantifier.pl script from MiRDeep2.

### Differential expression

Differential expression was calculated using the DESeq2 [69] package in the R programming language. Expression was calculated based on read counts that were normalized using DeSeq2 statistical program. DeSeq2 normalizes read counts for sequencing depth and RNA composition. Five different life stages were compared: Egg to Larva, Larva to Pupa, Pupa to Female, and Pupa to Male. DESeq2 utilized a negative binomial generalized linear model to test for statistical significance in expression between the life stages. Raw read counts are normalized using a median of ratios method which accounts for sequencing depth and RNA composition. Therefore, read counts were normalized for more than reads per million to account for differences in sequencing quality and length of microRNAs. All analysis was completed using R [70–72].

## Supporting information

S1 Table Conserved *H*. *illucens* miRNA identified in other arthropod species. miRNA provisionally named using *hil* for species and number taken from miRNA identified in other arthropod species. Identical locations collapsed and those with multiple locations included below the bold. If no other location is listed, all miRNA were found on the same location. *Life stage abbreviations: E = Egg; L = Larva; P = Pupa; F = Female; M = Male.
(DOCX)

S2 Table Unique *H*. *illucens* miRNA not identified in any other species. miRNA provisionally named; identical locations collapsed and those with multiple locations included below the bold. If no other location is listed, all miRNA were found on the same location.*Life stage abbreviations: E = Egg; L = Larva; P = Pupa; F = Female; M = Male.
(DOCX)

S3 Table Unique *H*. *illucens* miRNA log fold change (LFC) and P-adjusted value between life stages. miRNA provisionally named; Green filled boxes represent statistically significant up regulated LFC; Red filled boxes represent statistically significant down regulated LFC.
(DOCX)

S4 Table Conserved *H*. *illucens* miRNA log fold change (LFC) and DESeq2 P-adjusted value between life stages. miRNA provisionally named; Green filled boxes represent statistically significant up regulated LFC; Red filled boxes represent statistically significant down regulated LFC.
(DOCX)
