# Supplementary material for: microRNA profile of Hermetia illucens (black soldier fly) and its implications on mass rearing
Source: PLoS One. 2022 Mar 17;17(3):e0265492. doi: 10.1371/journal.pone.0265492 (PMC8929568; doi:10.1371/journal.pone.0265492)
Supplement: S4 Table — miRNA provisionally named; Green filled boxes represent statistically significant up regulated LFC; Red filled boxes represent statistically significant down regulated LFC. (DOCX) [file pone.0265492.s004.docx]

**S4 Table. Conserved *H. illucens* miRNA log fold change (LFC) and P-Adjusted Value between life stages.**miRNA provisionally named; Green filled boxes represent statistically significant up regulated LFC; Red filled boxes represent statistically significant down regulated LFC.

|  | Egg v Female | | Egg v Male | | Egg v Pupa | | Larva v Female | |
| --- | --- | --- | --- | --- | --- | --- | --- | --- |
| miRNA | LFC | P-Adjusted Value | LFC | P-Adjusted Value | LFC | P-Adjusted Value | LFC | P-Adjusted Value |
| hil-miR-11894a | 1.284 | NA | -0.038 | NA | -0.015 | NA | 1.186 | 0.831 |
| hil-miR-11903b | 1.668 | NA | -0.039 | NA | -0.016 | NA | 1.571 | 0.620 |
| hil-miR-11913 | -0.059 | NA | -0.039 | NA | 1.428 | NA | -0.155 | 0.930 |
| hil-miR-11927 | 1.818 | NA | -0.039 | NA | -0.016 | NA | 1.721 | 0.555 |
| hil-miR-263a-3p | -0.058 | NA | 1.306 | NA | -0.016 | NA | -0.155 | 0.930 |
| hil-miR-278-5p | -2.223 | NA | -2.203 | NA | -2.180 | NA | -0.155 | 0.930 |
| hil-miR-2940-3p | 1.668 | NA | -0.039 | NA | -0.016 | NA | 1.571 | 0.620 |
| hil-miR-2a-5p | -12.213 | 2.05E-21 | -12.192 | 2.27E-21 | -12.171 | 3.81E-21 | -1.735 | 0.445 |
| hil-miR-309b-5p | 2.020 | NA | -0.037 | NA | -0.015 | 0.995 | 1.922 | 0.470 |
| hil-miR-10357-5p | -0.059 | 0.977 | -0.039 | 0.983 | 2.886 | 0.121 | -0.155 | 0.930 |
| hil-miR-10361b-3p | -0.058 | 0.977 | -0.039 | 0.983 | 1.761 | 0.420 | -1.783 | 0.497 |
| hil-miR-10361b-5p | 1.949 | NA | -0.039 | NA | -0.016 | 0.995 | 1.852 | 0.497 |
| hil-miR-10371-5p | -0.058 | NA | 1.028 | NA | -0.016 | NA | -0.155 | NA |
| hil-miR-10373-3p | 2.230 | 0.230 | -0.037 | 0.983 | -0.015 | 0.995 | 2.133 | 0.399 |
| hil-miR-12418-3p | -0.057 | 0.977 | -0.036 | 0.983 | 6.269 | 7.01E-05 | -0.155 | 0.930 |
| hil-miR-998-5p | -12.360 | 8.57E-15 | -12.342 | 8.41E-15 | -4.573 | 0.001 | -2.482 | 0.312 |
| hil-miR-2788-3p | -0.059 | NA | -0.039 | NA | 1.428 | NA | -0.155 | 0.930 |
| hil-miR-3724-3p | 1.147 | NA | -0.039 | NA | -0.016 | NA | 1.050 | NA |
| hil-miR-3752-3p | -0.058 | 0.977 | -0.037 | 0.983 | -0.015 | 0.995 | -2.444 | 0.303 |
| hil-miR-3777-3p | -0.058 | NA | -0.038 | NA | 1.718 | NA | -0.155 | 0.930 |
| hil-miR-6042-3p | -6.645 | 3.22E-11 | -15.068 | 1.47E-40 | -14.911 | 1.59E-40 | 4.900 | 3.28E-06 |
| hil-miR-6055-5p | -4.273 | 0.007 | -4.254 | 0.007 | -4.232 | 0.010 | -0.155 | 0.930 |
| hil-miR-928-5p | 1.397 | NA | -0.038 | NA | -0.015 | NA | 1.300 | 0.763 |
| hil-miR-9878-3p | -0.057 | 0.977 | 6.916 | 9.32E-06 | -0.014 | 0.995 | -0.155 | 0.930 |
| hil-miR-9893-5p | -2.006 | NA | -1.987 | NA | -1.964 | 0.376 | -0.155 | 0.930 |
| hil-miR-263b-3p | -0.058 | NA | -0.039 | NA | 1.811 | NA | -0.155 | 0.930 |
| hil-miR-2744 | -0.059 | 0.977 | -0.039 | 0.983 | 2.348 | 0.243 | -0.155 | 0.930 |
| hil-miR-2774a | -0.058 | 0.977 | -0.040 | 0.983 | 7.056 | 4.46E-07 | -0.155 | 0.930 |
| hil-miR-2775b | -0.057 | 0.977 | -0.039 | 0.983 | 7.060 | 2.45E-08 | -0.155 | 0.930 |
| hil-miR-2780a-5p | 1.491 | NA | -0.039 | NA | -0.016 | NA | 1.393 | 0.702 |
| hil-miR-279d-5p | -0.059 | 0.977 | -0.039 | 0.983 | 3.869 | 0.020 | -0.155 | 0.930 |
| hil-miR-2801 | -6.941 | 3.30E-06 | -6.921 | 3.28E-06 | -6.898 | 4.91E-06 | -0.155 | 0.930 |
| hil-miR-2807a | -0.058 | NA | 1.511 | NA | -0.015 | NA | -0.155 | 0.930 |
| hil-miR-2838 | -0.058 | NA | -0.037 | NA | 1.936 | 0.385 | -0.155 | 0.930 |
| hil-miR-2856-3p | -0.058 | NA | 1.688 | NA | -0.015 | NA | -0.155 | 0.930 |
| hil-miR-2999 | -6.066 | 3.66E-05 | -6.047 | 3.80E-05 | -6.025 | 5.31E-05 | -0.155 | 0.930 |
| hil-miR-308-5p | -0.057 | 0.977 | -0.040 | 0.983 | 5.409 | 1.45E-04 | -0.155 | 0.930 |
| hil-miR-3212 | -0.058 | NA | -0.039 | NA | -0.016 | NA | -1.213 | NA |
| hil-miR-3213-3p | -1.800 | NA | -1.780 | NA | -1.757 | NA | -0.155 | 0.930 |
| hil-miR-3213-5p | -0.059 | 0.977 | -0.039 | 0.983 | 2.471 | 0.205 | -0.155 | 0.930 |
| hil-miR-3227 | -0.058 | 0.977 | 2.523 | 0.172 | -0.015 | 0.995 | -4.445 | 0.012 |
| hil-miR-3228 | -0.059 | NA | -0.039 | NA | 1.707 | NA | -0.155 | 0.930 |
| hil-miR-3238 | -0.058 | 0.977 | -0.037 | 0.983 | -0.015 | 0.995 | -4.505 | 0.003 |
| hil-miR-3264 | -0.866 | 0.779 | -3.057 | 0.097 | -3.034 | 0.122 | 2.056 | 0.452 |
| hil-miR-3265 | -1.395 | NA | -1.376 | NA | -1.353 | NA | -0.155 | 0.930 |
| hil-miR-3276 | -0.058 | 0.977 | -0.038 | 0.983 | 6.422 | 2.31E-05 | -6.572 | 1.55E-05 |
| hil-miR-3279 | -0.058 | 0.977 | 2.239 | 0.232 | -0.015 | 0.995 | -0.155 | 0.930 |
| hil-miR-3296-5p | -0.058 | 0.977 | 3.307 | 0.050 | -0.015 | 0.995 | -0.155 | 0.930 |
| hil-miR-3323 | -1.509 | NA | -1.489 | NA | -1.467 | NA | -0.155 | 0.930 |
| hil-miR-3338-5p | -0.058 | NA | 0.851 | NA | -0.016 | NA | -0.155 | NA |
| hil-miR-3375-3p | -0.058 | NA | -0.038 | NA | 1.438 | NA | -0.155 | 0.930 |
| hil-miR-3381-3p | -0.058 | 0.977 | 3.066 | 0.074 | -0.015 | 0.995 | -0.155 | 0.930 |
| hil-miR-140-5p | -0.058 | 0.977 | -0.039 | 0.983 | 4.419 | 0.004 | -0.155 | 0.930 |
| hil-miR-144-5p | 11.997 | 2.43E-13 | 11.672 | 9.14E-13 | 6.226 | 2.73E-04 | 11.901 | 7.64E-13 |
| hil-miR-146c-3p | 1.284 | NA | -0.038 | NA | -0.015 | NA | 1.186 | 0.831 |
| hil-miR-181b-3p | -0.058 | 0.977 | -0.039 | 0.983 | 7.803 | 3.73E-07 | -0.155 | 0.930 |
| hil-miR-223-3p | 0.889 | NA | 1.082 | NA | -0.015 | NA | 0.792 | 0.930 |
| hil-miR-490-5p | -0.058 | 0.977 | -0.037 | NA | -0.015 | 0.995 | -2.290 | 0.351 |
| hil-bantam-3p | 5.532 | 1.98E-12 | 5.373 | 7.47E-12 | 2.063 | 0.017 | 4.686 | 4.98E-09 |
| hil-let-7-5p | 10.850 | 4.09E-18 | 10.753 | 5.56E-18 | 8.807 | 3.78E-12 | 10.753 | 1.93E-17 |
| hil-miR-1000-5p | 4.809 | 3.02E-07 | 4.794 | 3.08E-07 | 2.565 | 0.011 | 8.307 | 5.81E-11 |
| hil-miR-1002-5p | -5.672 | 7.94E-11 | -6.687 | 1.64E-14 | -13.032 | 1.92E-26 | 4.270 | 9.32E-06 |
| hil-miR-100-5p | 7.973 | 3.45E-22 | 7.622 | 1.41E-20 | 6.524 | 3.45E-15 | 13.019 | 7.88E-28 |
| hil-miR-10-5p | 0.315 | 0.971 | -1.925 | 0.021 | -4.587 | 7.14E-09 | 3.509 | 1.27E-05 |
| hil-miR-124-3p | 1.706 | 0.273 | 2.229 | 0.145 | 0.901 | 0.806 | 6.315 | 1.59E-04 |
| hil-miR-125-5p | 9.790 | 1.73E-17 | 9.566 | 6.99E-17 | 8.720 | 7.55E-14 | 9.694 | 8.13E-17 |
| hil-miR-12-5p | 3.974 | 6.47E-05 | 2.771 | 0.007 | -0.827 | 0.634 | 5.942 | 1.95E-09 |
| hil-miR-133-3p | 8.875 | 2.59E-13 | 9.085 | 5.65E-14 | 5.523 | 1.11E-05 | 6.900 | 1.10E-08 |
| hil-miR-137-3p | 2.793 | 0.088 | -0.038 | 0.983 | -0.015 | 0.995 | 2.696 | 0.156 |
| hil-miR-1-3p | 5.300 | 4.24E-09 | 4.930 | 4.98E-08 | 1.541 | 0.157 | 6.589 | 5.42E-13 |
| hil-miR-14-3p | 5.525 | 1.48E-09 | 5.044 | 3.69E-08 | 2.202 | 0.030 | 5.776 | 4.37E-10 |
| hil-miR-14-5p | 0.021 | 0.986 | -4.075 | 0.008 | -4.054 | 0.011 | 3.961 | 0.015 |
| hil-miR-184-3p | -0.199 | 0.977 | -0.100 | 0.983 | -1.630 | 0.099 | 4.560 | 1.15E-07 |
| hil-miR-190-5p | -1.500 | 0.087 | -1.791 | 0.035 | -3.604 | 1.00E-05 | 3.376 | 8.19E-05 |
| hil-miR-193-3p | 7.732 | 6.68E-06 | 6.133 | 4.15E-04 | 6.602 | 1.68E-04 | 7.634 | 1.27E-05 |
| hil-miR-210-3p | 11.585 | 5.37E-21 | 11.442 | 1.42E-20 | 7.189 | 1.41E-08 | 11.488 | 3.02E-20 |
| hil-miR-2280-3p | 1.583 | NA | -0.039 | NA | -0.016 | NA | 1.486 | 0.656 |
| hil-miR-2492-5p | 1.276 | NA | -0.039 | NA | -0.016 | NA | 1.178 | NA |
| hil-miR-2499-3p | -1.536 | 0.443 | 1.155 | 0.587 | -1.499 | 0.545 | -1.440 | 0.654 |
| hil-miR-252-5p | 8.664 | 5.17E-13 | 8.250 | 5.62E-12 | 6.854 | 2.38E-08 | 5.416 | 3.34E-06 |
| hil-miR-263a-5p | 3.250 | 1.88E-04 | 2.715 | 0.002 | 2.189 | 0.018 | 5.546 | 1.13E-10 |
| hil-miR-263b-5p | 4.362 | 2.50E-05 | 3.709 | 3.77E-04 | 2.536 | 0.025 | 6.131 | 2.27E-06 |
| hil-miR-274-5p | 10.383 | 8.72E-18 | 10.214 | 1.91E-17 | 6.620 | 9.30E-08 | 10.286 | 3.87E-17 |
| hil-miR-275-3p | 0.969 | 0.843 | -0.117 | 0.983 | 2.088 | 0.416 | 1.789 | 0.616 |
| hil-miR-275-5p | 7.711 | 1.14E-04 | 9.863 | 4.90E-07 | -0.013 | 0.995 | 7.612 | 2.08E-04 |
| hil-miR-276a-3p | 6.500 | 1.05E-10 | 6.449 | 1.42E-10 | 4.491 | 1.63E-05 | 6.454 | 2.77E-10 |
| hil-miR-277-3p | 8.367 | 1.73E-17 | 8.402 | 9.55E-18 | 0.948 | 0.552 | 9.505 | 3.36E-21 |
| hil-miR-278-3p | 8.415 | 3.43E-11 | 8.534 | 1.59E-11 | 7.893 | 1.08E-09 | 2.478 | 0.018 |
| hil-miR-279-3p | 0.283 | 0.977 | -0.547 | 0.815 | -1.950 | 0.049 | 5.561 | 7.19E-10 |
| hil-miR-281-2-5p | 11.757 | 1.58E-28 | 11.819 | 4.88E-29 | 6.958 | 1.49E-10 | 4.312 | 1.00E-04 |
| hil-miR-282-5p | 6.600 | 1.83E-05 | 6.256 | 4.95E-05 | 7.425 | 1.50E-06 | 1.405 | 0.497 |
| hil-miR-283-5p | 2.080 | 0.013 | 1.299 | 0.153 | -0.938 | 0.390 | 2.481 | 0.004 |
| hil-miR-285-3p | -4.037 | 1.88E-04 | -4.244 | 7.93E-05 | -6.107 | 1.41E-08 | 2.992 | 0.011 |
| hil-miR-2a-3p | -0.046 | 0.977 | -0.346 | 0.983 | -1.700 | 0.171 | 5.466 | 2.00E-07 |
| hil-miR-304-5p | 3.100 | 1.94E-04 | 1.678 | 0.061 | -1.612 | 0.099 | 6.363 | 4.33E-13 |
| hil-miR-305-5p | 4.104 | 4.57E-05 | 3.225 | 0.002 | 3.074 | 0.004 | 2.620 | 0.017 |
| hil-miR-307a-3p | 4.979 | 5.15E-05 | 4.907 | 6.28E-05 | 4.444 | 3.83E-04 | 7.098 | 1.19E-07 |
| hil-miR-311-5p | -3.382 | 0.066 | -3.365 | 0.067 | -0.708 | 0.995 | -0.155 | 0.930 |
| hil-miR-314-5p | 1.668 | NA | -0.039 | NA | -0.016 | NA | 1.571 | 0.620 |
| hil-miR-315-5p | 1.252 | 0.364 | 0.628 | 0.887 | -0.288 | 0.995 | 5.435 | 3.83E-06 |
| hil-miR-316-5p | 8.351 | 9.89E-16 | 7.932 | 2.10E-14 | 7.616 | 5.11E-13 | 2.984 | 0.003 |
| hil-miR-317-3p | 0.431 | 0.977 | -9.952 | 5.61E-08 | -0.453 | 0.995 | 2.058 | 0.436 |
| hil-miR-317-5p | 6.072 | 0.001 | 11.392 | 1.48E-10 | -0.032 | 0.995 | 3.530 | 0.071 |
| hil-miR-31b-5p | 7.225 | 4.89E-17 | 7.525 | 2.23E-18 | 4.853 | 3.71E-08 | 4.452 | 5.59E-07 |
| hil-miR-33-5p | 2.155 | 0.020 | 1.405 | 0.159 | -0.815 | 0.561 | 4.403 | 7.80E-07 |
| hil-miR-3-3p | -4.610 | 6.55E-05 | -2.392 | 0.054 | -11.385 | 6.52E-18 | 7.335 | 5.59E-07 |
| hil-miR-34-3p | -2.557 | 0.163 | -2.537 | 0.160 | -2.513 | 0.195 | -0.155 | 0.930 |
| hil-miR-34-5p | 8.151 | 5.90E-08 | 6.775 | 8.30E-06 | 0.114 | 0.995 | 4.392 | 0.006 |
| hil-miR-375-3p | 7.067 | 2.05E-21 | 7.087 | 1.49E-21 | -0.010 | 0.995 | 1.957 | 0.018 |
| hil-miR-4-3p | -1.982 | 0.230 | -5.303 | 3.13E-04 | -5.169 | 0.001 | 5.695 | 3.09E-04 |
| hil-miR-4982-3p | -2.555 | 0.163 | -2.536 | 0.160 | -2.514 | 0.195 | -0.155 | 0.930 |
| hil-miR-4986-3p | 1.885 | NA | -0.039 | NA | -0.016 | NA | 1.788 | 0.518 |
| hil-miR-5-5p | 5.189 | 6.19E-05 | 6.476 | 3.29E-07 | -0.016 | 0.995 | 5.093 | 1.28E-04 |
| hil-miR-6-2-5p | -14.288 | 1.89E-32 | -14.267 | 1.09E-32 | -14.244 | 1.55E-32 | -0.155 | 0.930 |
| hil-miR-7-5p | -1.301 | 0.182 | -2.900 | 0.001 | -4.779 | 3.41E-08 | 8.363 | 2.44E-16 |
| hil-miR-8-3p | 5.139 | 5.03E-06 | 4.271 | 1.66E-04 | 2.507 | 0.045 | 4.311 | 2.15E-04 |
| hil-miR-87-3p | 4.859 | 1.88E-04 | 4.540 | 4.99E-04 | 1.941 | 0.249 | 4.762 | 3.77E-04 |
| hil-miR-927-5p | 10.013 | 4.47E-17 | 10.110 | 1.73E-17 | 6.380 | 1.90E-07 | 8.569 | 8.77E-18 |
| hil-miR-929-3p | -0.059 | 0.977 | 2.387 | 0.192 | -0.015 | 0.995 | -0.155 | 0.930 |
| hil-miR-929-5p | 4.962 | 0.003 | 5.001 | 0.003 | 2.256 | 0.293 | 4.864 | 0.006 |
| hil-miR-92a-3p | -3.290 | 0.002 | -4.976 | 2.07E-06 | -4.670 | 0.000 | 6.275 | 4.86E-09 |
| hil-miR-932-5p | 3.346 | 0.006 | 3.920 | 0.001 | 3.287 | 0.010 | 6.889 | 3.43E-06 |
| hil-miR-9378-5p | -4.130 | 0.006 | -6.329 | 1.39E-04 | -6.307 | 1.78E-04 | 2.066 | 0.452 |
| hil-miR-9385-5p | -0.058 | NA | -0.039 | NA | -0.016 | NA | -1.705 | 0.568 |
| hil-miR-956-3p | -2.566 | 0.163 | -2.546 | 0.160 | 5.103 | 2.32E-04 | -0.155 | 0.930 |
| hil-miR-957-3p | 8.177 | 0.000 | 8.636 | 4.14E-18 | 4.234 | 5.59E-05 | 9.231 | 2.02E-14 |
| hil-miR-958-3p | 10.310 | 5.24E-13 | 9.415 | 4.32E-11 | 5.638 | 1.68E-04 | 0.626 | 0.930 |
| hil-miR-961-5p | -3.380 | 0.006 | -5.406 | 1.30E-04 | -5.384 | 1.68E-04 | 1.887 | 0.412 |
| hil-miR-965-3p | 0.941 | 0.664 | 0.352 | 0.983 | -0.981 | 0.731 | 6.633 | 5.75E-06 |
| hil-miR-970-3p | 4.801 | 2.75E-06 | 4.412 | 1.71E-05 | 1.069 | 0.471 | 6.993 | 1.73E-11 |
| hil-miR-971-3p | 4.046 | 0.005 | 4.451 | 0.002 | 1.367 | 0.563 | 3.948 | 0.009 |
| hil-miR-976-5p | -0.058 | NA | -0.039 | NA | -0.015 | NA | -1.502 | 0.656 |
| hil-miR-977-3p | 1.607 | NA | 1.288 | NA | -0.016 | NA | 1.510 | 0.656 |
| hil-miR-980-3p | 5.567 | 1.34E-06 | 5.233 | 5.64E-06 | 3.216 | 0.009 | 6.024 | 3.13E-07 |
| hil-miR-981-3p | 5.449 | 7.54E-06 | 5.023 | 4.09E-05 | 4.795 | 1.18E-04 | 7.582 | 2.01E-07 |
| hil-miR-988-3p | 6.979 | 1.64E-06 | 5.313 | 3.30E-04 | 5.699 | 1.41E-04 | 6.881 | 3.43E-06 |
| hil-miR-988-5p | -7.871 | 1.16E-08 | -4.983 | 9.82E-06 | -7.827 | 2.38E-08 | -0.155 | 0.930 |
| hil-miR-993-3p | -4.587 | 6.19E-05 | -4.097 | 3.49E-04 | -7.472 | 1.09E-09 | 4.495 | 0.003 |
| hil-miR-993-5p | 2.367 | 0.211 | -0.038 | 0.983 | 2.950 | 0.123 | 2.270 | 0.363 |
| hil-miR-999-3p | 7.117 | 2.34E-12 | 7.095 | 2.53E-12 | 4.488 | 2.00E-05 | 6.306 | 9.53E-10 |
| hil-miR-9a-3p | 4.049 | 0.021 | 3.013 | 0.100 | 2.679 | 0.184 | 6.529 | 0.001 |
| hil-miR-9a-5p | -1.669 | 0.092 | -2.243 | 0.017 | -2.907 | 0.002 | 2.984 | 0.002 |
| hil-miR-iab-4-5p | 1.442 | 0.273 | -1.543 | 0.253 | -1.981 | 0.151 | 7.592 | 2.00E-07 |
| hil-miR-iab-8-5p | -0.058 | 0.977 | 7.158 | 7.78E-09 | -0.017 | 0.995 | -0.155 | 0.930 |
| hil-miR-193-5p | -0.058 | NA | -0.038 | NA | -0.015 | NA | -1.807 | 0.518 |
| hil-miR-2548-3p | 1.759 | 0.337 | -0.038 | 0.983 | -0.015 | 0.995 | -1.963 | 0.303 |
| hil-miR-311-5p | -0.058 | 0.977 | -0.038 | 0.983 | 6.424 | 6.24E-05 | -8.258 | 1.86E-07 |
| hil-miR-iab-8-3p | -1.790 | NA | -1.770 | NA | -1.746 | NA | -0.155 | 0.930 |
| hil-miR-973-5p | -0.060 | 0.977 | 6.256 | 4.31E-05 | 1.381 | 0.622 | -0.155 | 0.930 |
| hil-miR-2498b-5p | -0.058 | 0.977 | -0.037 | 0.983 | 3.706 | 0.018 | -0.155 | 0.930 |
| hil-miR-2765 | 4.998 | 5.47E-06 | 2.117 | 0.079 | 5.772 | 1.61E-07 | 9.897 | 1.29E-12 |
| hil-miR-2767 | -1.611 | NA | -1.591 | NA | -1.569 | NA | -0.155 | 0.930 |
| hil-miR-2796 | 3.390 | 0.003 | 3.890 | 0.001 | 1.268 | 0.416 | 6.429 | 5.92E-06 |
| hil-miR-2a | -2.264 | 0.030 | -2.309 | 0.026 | -1.338 | 0.279 | 1.781 | 0.159 |
| hil-miR-3338 | 2.484 | 0.175 | -0.037 | 0.983 | -0.015 | 0.995 | 2.387 | 0.312 |
| hil-miR-6098 | 6.659 | 6.68E-06 | 5.988 | 5.54E-05 | 4.151 | 0.009 | 6.562 | 1.27E-05 |
| hil-miR-750 | 2.762 | 0.102 | -0.038 | 0.983 | -0.015 | 0.995 | 2.665 | 0.184 |
| hil-miR-219-3p | 2.083 | 0.272 | -0.039 | NA | -0.015 | 0.995 | 1.986 | 0.453 |
| hil-miR-3805a-5p | -7.278 | 1.90E-11 | -12.416 | 1.88E-19 | -12.393 | 3.94E-19 | 5.001 | 0.001 |
| hil-miR-3839-3p | -7.311 | 1.15E-07 | -7.291 | 1.15E-07 | -7.265 | 1.87E-07 | -0.155 | 0.930 |
| hil-miR-3844-3p | -1.867 | NA | -1.847 | NA | -1.824 | NA | -0.155 | 0.930 |
| hil-miR-3851o-2-5p | -1.612 | NA | -1.592 | NA | -1.568 | NA | -0.155 | 0.930 |
| hil-miR-3859-5p | -0.058 | 0.977 | 4.348 | 0.004 | 3.996 | 0.011 | -0.155 | 0.930 |
| hil-miR-3861-5p | -0.058 | NA | -0.038 | NA | 1.324 | NA | -0.155 | 0.930 |
| hil-miR-3862-3p | -0.057 | 0.977 | -0.039 | 0.983 | 5.925 | 6.81E-06 | -0.155 | 0.930 |
| hil-miR-3884-5p | -0.059 | 0.977 | -0.039 | 0.983 | -0.015 | 0.995 | -3.662 | 0.037 |
| hil-miR-6011-5p | 0.236 | 0.977 | -6.291 | 5.82E-05 | -4.125 | 0.004 | 6.391 | 6.74E-05 |

|  | Egg v Larva | | Larva v Pupa | | Pupa v Female | | Pupa v Male | |
| --- | --- | --- | --- | --- | --- | --- | --- | --- |
| miRNA | LFC | P-Adjusted Value | LFC | P-Adjusted Value | LFC | P-Adjusted Value | LFC | P-Adjusted Value |
| hil-miR-11894a | -0.098 | NA | 0.112 | NA | 1.298 | NA | -0.023 | NA |
| hil-miR-11903b | -0.097 | NA | 0.114 | NA | 1.684 | NA | -0.023 | NA |
| hil-miR-11913 | -0.097 | NA | -1.331 | NA | -1.487 | NA | -1.467 | NA |
| hil-miR-11927 | -0.097 | NA | 0.114 | NA | 1.834 | NA | -0.023 | NA |
| hil-miR-263a-3p | -0.097 | NA | 0.113 | NA | -0.042 | NA | 1.322 | NA |
| hil-miR-278-5p | 2.068 | NA | 0.113 | NA | -0.043 | NA | -0.023 | NA |
| hil-miR-2940-3p | -0.097 | NA | 0.114 | NA | 1.684 | NA | -0.023 | NA |
| hil-miR-2a-5p | 10.479 | 1.82E-22 | 1.692 | 0.488 | -0.043 | 0.981 | -0.022 | 0.990 |
| hil-miR-309b-5p | -0.098 | 0.962 | 0.112 | 0.957 | 2.035 | NA | -0.023 | NA |
| hil-miR-10357-5p | -0.097 | 0.962 | -2.789 | 0.189 | -2.945 | 0.108 | -2.925 | 0.129 |
| hil-miR-10361b-3p | -1.725 | 0.518 | -0.036 | 0.979 | -1.818 | 0.396 | -1.800 | 0.474 |
| hil-miR-10361b-5p | -0.097 | 0.962 | 0.114 | NA | 1.966 | NA | -0.023 | NA |
| hil-miR-10371-5p | -0.097 | NA | 0.113 | NA | -0.042 | NA | 1.043 | NA |
| hil-miR-10373-3p | -0.098 | 0.962 | 0.112 | 0.957 | 2.245 | NA | -0.023 | NA |
| hil-miR-12418-3p | -0.098 | 0.962 | -6.171 | 1.80E-04 | -6.326 | 7.75E-05 | -6.305 | 7.80E-05 |
| hil-miR-998-5p | 9.878 | 2.88E-12 | -5.306 | 0.001 | -7.787 | 2.98E-06 | -7.769 | 2.98E-06 |
| hil-miR-2788-3p | -0.097 | NA | -1.331 | NA | -1.487 | NA | -1.467 | NA |
| hil-miR-3724-3p | -0.097 | NA | 0.113 | NA | 1.163 | NA | -0.023 | NA |
| hil-miR-3752-3p | -2.387 | 0.278 | 2.401 | 0.310 | -0.043 | 0.981 | -0.023 | NA |
| hil-miR-3777-3p | -0.098 | NA | -1.620 | NA | -1.776 | NA | -1.756 | NA |
| hil-miR-6042-3p | 11.544 | 1.93E-30 | 3.367 | 0.010 | 8.267 | 1.23E-12 | -0.157 | 0.990 |
| hil-miR-6055-5p | 4.117 | 0.015 | 0.114 | 0.957 | -0.041 | 0.981 | -0.022 | 0.990 |
| hil-miR-928-5p | -0.098 | NA | 0.112 | NA | 1.412 | NA | -0.023 | NA |
| hil-miR-9878-3p | -0.098 | 0.962 | 0.112 | 0.957 | -0.043 | 0.981 | 6.930 | 1.54E-05 |
| hil-miR-9893-5p | 1.851 | 0.492 | 0.114 | NA | -0.041 | NA | -0.023 | NA |
| hil-miR-263b-3p | -0.097 | NA | -1.714 | NA | -1.869 | NA | -1.850 | NA |
| hil-miR-2744 | -0.097 | 0.962 | -2.251 | 0.369 | -2.407 | NA | -2.387 | NA |
| hil-miR-2774a | -0.098 | 0.962 | -6.958 | 1.64E-06 | -7.114 | 5.81E-07 | -7.096 | 5.93E-07 |
| hil-miR-2775b | -0.098 | 0.962 | -6.962 | 1.08E-07 | -7.118 | 2.79E-08 | -7.099 | 3.21E-08 |
| hil-miR-2780a-5p | -0.097 | NA | 0.114 | NA | 1.507 | NA | -0.023 | NA |
| hil-miR-279d-5p | -0.097 | 0.962 | -3.772 | 0.035 | -3.928 | 0.020 | -3.907 | 0.024 |
| hil-miR-2801 | 6.786 | 8.47E-06 | 0.113 | 0.957 | -0.043 | 0.981 | -0.023 | 0.990 |
| hil-miR-2807a | -0.098 | NA | 0.112 | NA | -0.043 | NA | 1.526 | NA |
| hil-miR-2838 | -0.098 | 0.962 | -1.839 | NA | -1.994 | NA | -1.974 | NA |
| hil-miR-2856-3p | -0.098 | NA | 0.112 | NA | -0.043 | NA | 1.703 | NA |
| hil-miR-2999 | 5.911 | 9.03E-05 | 0.114 | 0.957 | -0.041 | 0.981 | -0.022 | 0.990 |
| hil-miR-308-5p | -0.098 | 0.962 | -5.311 | 3.64E-04 | -5.466 | 1.47E-04 | -5.449 | 1.58E-04 |
| hil-miR-3212 | -1.155 | NA | 1.170 | NA | -0.042 | NA | -0.023 | NA |
| hil-miR-3213-3p | 1.645 | NA | 0.112 | NA | -0.043 | NA | -0.023 | NA |
| hil-miR-3213-5p | -0.097 | 0.962 | -2.375 | 0.313 | -2.530 | 0.192 | -2.510 | NA |
| hil-miR-3227 | -4.387 | 0.014 | 4.402 | 0.016 | -0.043 | 0.981 | 2.538 | 0.228 |
| hil-miR-3228 | -0.097 | NA | -1.610 | NA | -1.765 | NA | -1.745 | NA |
| hil-miR-3238 | -4.448 | 0.004 | 4.462 | 0.005 | -0.043 | 0.981 | -0.023 | 0.990 |
| hil-miR-3264 | 2.922 | 0.170 | 0.112 | 0.957 | 2.168 | 0.315 | -0.023 | 0.990 |
| hil-miR-3265 | 1.240 | NA | 0.113 | NA | -0.042 | NA | -0.023 | NA |
| hil-miR-3276 | -6.514 | 2.10E-05 | 0.092 | 0.957 | -6.480 | 2.60E-05 | -6.460 | 2.54E-05 |
| hil-miR-3279 | -0.098 | 0.962 | 0.112 | 0.957 | -0.043 | NA | 2.254 | NA |
| hil-miR-3296-5p | -0.098 | 0.962 | 0.112 | 0.957 | -0.043 | 0.981 | 3.322 | 0.068 |
| hil-miR-3323 | 1.353 | NA | 0.113 | NA | -0.042 | NA | -0.023 | NA |
| hil-miR-3338-5p | -0.097 | NA | 0.113 | NA | -0.042 | NA | 0.867 | NA |
| hil-miR-3375-3p | -0.098 | NA | -1.341 | NA | -1.496 | NA | -1.476 | NA |
| hil-miR-3381-3p | -0.098 | 0.962 | 0.112 | 0.957 | -0.043 | 0.981 | 3.081 | 0.102 |
| hil-miR-140-5p | -0.097 | 0.962 | -4.322 | 0.007 | -4.477 | 0.003 | -4.458 | 0.004 |
| hil-miR-144-5p | -0.096 | 0.962 | -6.130 | 0.001 | 5.771 | 8.20E-05 | 5.446 | 2.12E-04 |
| hil-miR-146c-3p | -0.098 | NA | 0.112 | NA | 1.298 | NA | -0.023 | NA |
| hil-miR-181b-3p | -0.097 | 0.962 | -7.705 | 1.44E-06 | -7.861 | 5.01E-07 | -7.841 | 5.31E-07 |
| hil-miR-223-3p | -0.097 | NA | 0.113 | NA | 0.905 | NA | 1.097 | NA |
| hil-miR-490-5p | -2.233 | 0.332 | 2.248 | 0.369 | -0.043 | NA | -0.023 | NA |
| hil-bantam-3p | -0.846 | 0.536 | -1.217 | 0.306 | 3.469 | 2.71E-05 | 3.311 | 6.33E-05 |
| hil-let-7-5p | -0.097 | 0.962 | -8.710 | 1.69E-11 | 2.043 | 0.049 | 1.946 | 0.070 |
| hil-miR-1000-5p | 3.499 | 0.015 | -6.064 | 4.70E-06 | 2.243 | 0.026 | 2.228 | 0.031 |
| hil-miR-1002-5p | 9.942 | 2.95E-27 | 3.090 | 0.042 | 7.360 | 7.09E-09 | 6.345 | 7.12E-07 |
| hil-miR-100-5p | 5.046 | 6.35E-05 | -11.570 | 7.01E-22 | 1.449 | 0.137 | 1.098 | 0.344 |
| hil-miR-10-5p | 3.194 | 9.24E-05 | 1.393 | 0.189 | 4.902 | 0.000 | 2.662 | 0.002 |
| hil-miR-124-3p | 4.609 | 0.010 | -5.510 | 0.002 | 0.805 | 0.868 | 1.328 | 0.596 |
| hil-miR-125-5p | -0.096 | 0.962 | -8.624 | 5.44E-13 | 1.070 | 0.268 | 0.846 | 0.507 |
| hil-miR-12-5p | 1.967 | 0.099 | -1.140 | 0.528 | 4.802 | 2.01E-06 | 3.598 | 0.001 |
| hil-miR-133-3p | -1.975 | 0.237 | -3.548 | 0.008 | 3.352 | 0.009 | 3.562 | 0.006 |
| hil-miR-137-3p | -0.097 | 0.962 | 0.112 | 0.957 | 2.809 | NA | -0.023 | NA |
| hil-miR-1-3p | 1.289 | 0.316 | -2.830 | 0.005 | 3.759 | 7.24E-05 | 3.389 | 3.90E-04 |
| hil-miR-14-3p | 0.251 | 0.962 | -2.453 | 0.020 | 3.323 | 0.001 | 2.842 | 0.005 |
| hil-miR-14-5p | 3.940 | 0.016 | 0.114 | 0.957 | 4.075 | 0.011 | -0.020 | 0.990 |
| hil-miR-184-3p | 4.759 | 2.72E-08 | -3.129 | 0.001 | 1.430 | 0.163 | 1.530 | 0.142 |
| hil-miR-190-5p | 4.877 | 4.30E-09 | -1.272 | 0.306 | 2.104 | 0.017 | 1.814 | 0.053 |
| hil-miR-193-3p | -0.098 | 0.962 | -6.504 | 3.97E-04 | 1.129 | 0.700 | -0.470 | 0.990 |
| hil-miR-210-3p | -0.097 | 0.962 | -7.092 | 5.85E-08 | 4.396 | 1.72E-06 | 4.253 | 3.44E-06 |
| hil-miR-2280-3p | -0.097 | NA | 0.114 | NA | 1.600 | NA | -0.023 | NA |
| hil-miR-2492-5p | -0.097 | NA | 0.113 | NA | 1.292 | NA | -0.023 | NA |
| hil-miR-2499-3p | 0.097 | 0.962 | 1.402 | 0.812 | -0.038 | 0.981 | 2.654 | 0.157 |
| hil-miR-252-5p | -3.248 | 0.017 | -3.605 | 0.004 | 1.810 | 0.189 | 1.397 | 0.407 |
| hil-miR-263a-5p | 2.296 | 0.016 | -4.485 | 5.67E-07 | 1.061 | 0.343 | 0.526 | 0.990 |
| hil-miR-263b-5p | 1.769 | 0.359 | -4.305 | 0.002 | 1.826 | 0.092 | 1.173 | 0.396 |
| hil-miR-274-5p | -0.097 | 0.962 | -6.523 | 4.10E-07 | 3.763 | 2.42E-05 | 3.594 | 5.62E-05 |
| hil-miR-275-3p | 0.821 | 0.962 | -2.908 | 0.281 | -1.119 | 0.866 | -2.205 | 0.438 |
| hil-miR-275-5p | -0.099 | 0.962 | 0.113 | 0.959 | 7.724 | 1.63E-04 | 9.876 | 0.000 |
| hil-miR-276a-3p | -0.046 | 0.962 | -4.446 | 3.57E-05 | 2.009 | 0.090 | 1.958 | 0.114 |
| hil-miR-277-3p | 1.138 | 0.496 | -2.086 | 0.099 | 7.419 | 2.34E-13 | 7.454 | 1.22E-13 |
| hil-miR-278-3p | -5.937 | 7.63E-06 | -1.956 | 0.097 | 0.522 | 0.903 | 0.641 | 0.914 |
| hil-miR-279-3p | 5.278 | 5.62E-09 | -3.328 | 0.001 | 2.233 | 0.022 | 1.402 | 0.217 |
| hil-miR-281-2-5p | -7.444 | 5.17E-12 | 0.486 | 0.957 | 4.798 | 1.47E-05 | 4.861 | 1.02E-05 |
| hil-miR-282-5p | -5.196 | 0.001 | -2.230 | 0.189 | -0.825 | 0.807 | -1.169 | 0.640 |
| hil-miR-283-5p | 0.402 | 0.962 | 0.537 | 0.957 | 3.018 | 3.18E-04 | 2.237 | 0.012 |
| hil-miR-285-3p | 7.029 | 5.72E-11 | -0.922 | 0.841 | 2.070 | 0.095 | 1.863 | 0.159 |
| hil-miR-2a-3p | 5.512 | 1.56E-07 | -3.812 | 0.001 | 1.654 | 0.189 | 1.353 | 0.356 |
| hil-miR-304-5p | 3.263 | 4.17E-04 | -1.651 | 0.165 | 4.712 | 2.79E-08 | 3.290 | 1.56E-04 |
| hil-miR-305-5p | -1.484 | 0.274 | -1.589 | 0.267 | 1.030 | 0.478 | 0.151 | 0.990 |
| hil-miR-307a-3p | 2.119 | 0.246 | -6.563 | 2.09E-06 | 0.534 | 0.981 | 0.463 | 0.990 |
| hil-miR-311-5p | 3.227 | 0.116 | -2.519 | 0.310 | -2.675 | 0.192 | -2.658 | 0.219 |
| hil-miR-314-5p | -0.097 | NA | 0.114 | NA | 1.684 | NA | -0.023 | NA |
| hil-miR-315-5p | 4.183 | 0.001 | -3.895 | 0.002 | 1.540 | 0.286 | 0.916 | 0.783 |
| hil-miR-316-5p | -5.367 | 5.95E-07 | -2.249 | 0.042 | 0.735 | 0.693 | 0.317 | 0.990 |
| hil-miR-317-3p | 1.627 | 0.594 | -1.174 | 0.957 | 0.884 | 0.914 | -9.499 | 5.31E-07 |
| hil-miR-317-5p | -2.543 | 0.332 | 2.575 | 0.369 | 6.105 | 0.001 | 11.424 | 4.52E-10 |
| hil-miR-31b-5p | -2.773 | 0.003 | -2.080 | 0.043 | 2.372 | 0.013 | 2.672 | 0.005 |
| hil-miR-33-5p | 2.247 | 0.022 | -1.433 | 0.250 | 2.970 | 0.001 | 2.219 | 0.025 |
| hil-miR-3-3p | 11.946 | 4.53E-17 | -0.561 | 0.957 | 6.775 | 8.09E-07 | 8.993 | 3.33E-11 |
| hil-miR-34-3p | 2.402 | 0.274 | 0.111 | 0.957 | -0.044 | 0.981 | -0.024 | NA |
| hil-miR-34-5p | -3.759 | 0.027 | 3.645 | 0.041 | 8.037 | 2.00E-07 | 6.661 | 1.86E-05 |
| hil-miR-375-3p | -5.110 | 1.10E-11 | 5.120 | 2.50E-11 | 7.077 | 6.51E-21 | 7.097 | 4.61E-21 |
| hil-miR-4-3p | 7.677 | 5.24E-07 | -2.508 | 0.244 | 3.188 | 0.051 | -0.134 | 0.990 |
| hil-miR-4982-3p | 2.400 | 0.274 | 0.114 | 0.957 | -0.041 | 0.981 | -0.022 | NA |
| hil-miR-4986-3p | -0.097 | NA | 0.114 | NA | 1.902 | NA | -0.023 | NA |
| hil-miR-5-5p | -0.097 | 0.962 | 0.113 | 0.957 | 5.205 | 8.99E-05 | 6.492 | 6.14E-07 |
| hil-miR-6-2-5p | 14.133 | 1.03E-31 | 0.111 | 0.957 | -0.044 | 0.981 | -0.023 | 0.990 |
| hil-miR-7-5p | 9.664 | 1.04E-21 | -4.885 | 5.77E-06 | 3.478 | 9.98E-05 | 1.880 | 0.063 |
| hil-miR-8-3p | -0.827 | 0.863 | -1.680 | 0.309 | 2.631 | 0.035 | 1.764 | 0.219 |
| hil-miR-87-3p | -0.097 | 0.962 | -1.844 | 0.369 | 2.918 | 0.009 | 2.599 | 0.026 |
| hil-miR-927-5p | -1.444 | 0.519 | -4.936 | 2.55E-06 | 3.633 | 2.81E-05 | 3.730 | 1.59E-05 |
| hil-miR-929-3p | -0.097 | 0.962 | 0.111 | 0.957 | -0.044 | 0.981 | 2.401 | NA |
| hil-miR-929-5p | -0.097 | 0.962 | -2.158 | 0.428 | 2.706 | 0.107 | 2.745 | 0.115 |
| hil-miR-92a-3p | 9.565 | 9.47E-20 | -4.895 | 1.21E-05 | 1.380 | 0.302 | -0.306 | 0.990 |
| hil-miR-932-5p | 3.543 | 0.034 | -6.830 | 6.91E-06 | 0.059 | 0.981 | 0.632 | 0.990 |
| hil-miR-9378-5p | 6.196 | 3.06E-04 | 0.110 | 0.957 | 2.176 | 0.315 | -0.023 | 0.990 |
| hil-miR-9385-5p | -1.647 | NA | 1.663 | NA | -0.042 | NA | -0.023 | NA |
| hil-miR-956-3p | 2.411 | 0.274 | -7.514 | 4.66E-06 | -7.669 | 1.96E-06 | -7.649 | 1.91E-06 |
| hil-miR-957-3p | 1.054 | 0.815 | -5.289 | 3.57E-05 | 3.942 | 4.57E-06 | 4.402 | 2.84E-07 |
| hil-miR-958-3p | -9.684 | 2.18E-11 | 4.046 | 0.001 | 4.672 | 9.98E-05 | 3.777 | 0.002 |
| hil-miR-961-5p | 5.267 | 3.09E-04 | 0.118 | 0.957 | 2.005 | 0.274 | -0.021 | 0.990 |
| hil-miR-965-3p | 5.692 | 1.33E-04 | -4.711 | 0.003 | 1.922 | 0.241 | 1.332 | 0.590 |
| hil-miR-970-3p | 2.193 | 0.076 | -3.261 | 0.005 | 3.732 | 4.62E-04 | 3.343 | 0.002 |
| hil-miR-971-3p | -0.097 | 0.962 | -1.269 | 0.841 | 2.679 | 0.050 | 3.085 | 0.023 |
| hil-miR-976-5p | -1.443 | NA | 1.458 | NA | -0.044 | NA | -0.024 | NA |
| hil-miR-977-3p | -0.097 | NA | 0.113 | NA | 1.623 | NA | 1.303 | NA |
| hil-miR-980-3p | 0.457 | 0.962 | -3.673 | 0.004 | 2.351 | 0.066 | 2.017 | 0.142 |
| hil-miR-981-3p | 2.133 | 0.308 | -6.927 | 4.52E-06 | 0.654 | 0.888 | 0.229 | 0.990 |
| hil-miR-988-3p | -0.097 | 0.962 | -5.602 | 3.53E-04 | 1.280 | 0.427 | -0.386 | 0.990 |
| hil-miR-988-5p | 7.716 | 3.98E-08 | 0.112 | 0.957 | -0.044 | 0.981 | 2.844 | 0.099 |
| hil-miR-993-3p | 9.082 | 1.80E-10 | -1.610 | 0.607 | 2.885 | 0.036 | 3.376 | 0.014 |
| hil-miR-993-5p | -0.097 | 0.962 | -2.853 | 0.195 | -0.583 | 0.981 | -2.988 | 0.135 |
| hil-miR-999-3p | -0.811 | 0.815 | -3.678 | 0.001 | 2.629 | 0.020 | 2.606 | 0.025 |
| hil-miR-9a-3p | 2.480 | 0.358 | -5.160 | 0.013 | 1.369 | 0.637 | 0.334 | 0.990 |
| hil-miR-9a-5p | 4.653 | 2.43E-07 | -1.746 | 0.125 | 1.237 | 0.268 | 0.664 | 0.843 |
| hil-miR-iab-4-5p | 6.150 | 3.87E-05 | -4.168 | 0.011 | 3.423 | 0.006 | 0.439 | 0.990 |
| hil-miR-iab-8-5p | -0.097 | 0.962 | 0.114 | 0.957 | -0.041 | 0.981 | 7.176 | 2.00E-08 |
| hil-miR-193-5p | -1.749 | NA | 1.764 | NA | -0.043 | NA | -0.023 | NA |
| hil-miR-2548-3p | -3.722 | 0.025 | 3.737 | 0.030 | 1.775 | 0.396 | -0.023 | 0.990 |
| hil-miR-311-5p | -8.199 | 2.20E-07 | 1.775 | 0.369 | -6.482 | 6.88E-05 | -6.463 | 6.70E-05 |
| hil-miR-iab-8-3p | 1.634 | NA | 0.112 | NA | -0.044 | NA | -0.024 | NA |
| hil-miR-973-5p | -0.096 | 0.962 | -1.285 | 0.909 | -1.440 | 0.596 | 4.875 | 0.001 |
| hil-miR-2498b-5p | -0.098 | 0.962 | -3.608 | 0.033 | -3.763 | 0.018 | -3.743 | 0.023 |
| hil-miR-2765 | 4.899 | 0.001 | -10.671 | 7.49E-14 | -0.774 | 0.755 | -3.655 | 0.002 |
| hil-miR-2767 | 1.455 | NA | 0.114 | NA | -0.042 | NA | -0.023 | NA |
| hil-miR-2796 | 3.039 | 0.069 | -4.307 | 0.005 | 2.122 | 0.093 | 2.622 | 0.034 |
| hil-miR-2a | 4.044 | 6.81E-05 | -2.706 | 0.015 | -0.926 | 0.550 | -0.971 | 0.595 |
| hil-miR-3338 | -0.098 | 0.962 | 0.112 | 0.957 | 2.499 | 0.198 | -0.023 | NA |
| hil-miR-6098 | -0.097 | 0.962 | -4.054 | 0.016 | 2.507 | 0.065 | 1.837 | 0.231 |
| hil-miR-750 | -0.097 | 0.962 | 0.112 | 0.957 | 2.777 | NA | -0.023 | NA |
| hil-miR-219-3p | -0.097 | 0.962 | 0.111 | 0.957 | 2.097 | NA | -0.024 | NA |
| hil-miR-3805a-5p | 12.279 | 6.27E-19 | 0.114 | 0.957 | 5.115 | 0.001 | -0.023 | 0.990 |
| hil-miR-3839-3p | 7.156 | 3.32E-07 | 0.110 | 0.957 | -0.046 | 0.981 | -0.025 | 0.990 |
| hil-miR-3844-3p | 1.712 | NA | 0.112 | NA | -0.044 | NA | -0.024 | NA |
| hil-miR-3851o-2-5p | 1.457 | NA | 0.112 | NA | -0.044 | NA | -0.024 | NA |
| hil-miR-3859-5p | -0.097 | 0.962 | -3.899 | 0.020 | -4.054 | 0.011 | 0.352 | 0.990 |
| hil-miR-3861-5p | -0.098 | NA | -1.227 | NA | -1.382 | NA | -1.362 | NA |
| hil-miR-3862-3p | -0.098 | 0.962 | -5.827 | 1.71E-05 | -5.982 | 7.30E-06 | -5.963 | 7.43E-06 |
| hil-miR-3884-5p | -3.603 | 0.040 | 3.618 | 0.045 | -0.044 | 0.981 | -0.024 | 0.990 |
| hil-miR-6011-5p | 6.155 | 1.33E-04 | -2.030 | 0.439 | 4.361 | 0.002 | -2.166 | 0.338 |

|  | Larva v Male | | Female v Male | |
| --- | --- | --- | --- | --- |
| miRNA | LFC | P-Adjusted Value | LFC | P-Adjusted Value |
| hil-miR-11894a | -0.135 | NA | -1.321 | NA |
| hil-miR-11903b | -0.136 | NA | -1.707 | NA |
| hil-miR-11913 | -0.135 | NA | 0.020 | NA |
| hil-miR-11927 | -0.136 | NA | -1.857 | NA |
| hil-miR-263a-3p | 1.209 | NA | 1.364 | NA |
| hil-miR-278-5p | -0.136 | NA | 0.020 | NA |
| hil-miR-2940-3p | -0.136 | NA | -1.707 | NA |
| hil-miR-2a-5p | -1.714 | 0.411 | 0.021 | 0.992 |
| hil-miR-309b-5p | -0.135 | NA | -2.057 | NA |
| hil-miR-10357-5p | -0.135 | 0.939 | 0.020 | 0.992 |
| hil-miR-10361b-3p | -1.764 | 0.496 | 0.019 | 0.992 |
| hil-miR-10361b-5p | -0.136 | NA | -1.988 | NA |
| hil-miR-10371-5p | 0.930 | NA | 1.086 | NA |
| hil-miR-10373-3p | -0.135 | NA | -2.268 | NA |
| hil-miR-12418-3p | -0.134 | 0.939 | 0.021 | 0.992 |
| hil-miR-998-5p | -2.463 | 0.252 | 0.018 | 0.992 |
| hil-miR-2788-3p | -0.135 | NA | 0.020 | NA |
| hil-miR-3724-3p | -0.136 | NA | -1.186 | NA |
| hil-miR-3752-3p | -2.424 | NA | 0.020 | NA |
| hil-miR-3777-3p | -0.135 | NA | 0.020 | NA |
| hil-miR-6042-3p | -3.524 | 0.004 | -8.424 | 7.34E-13 |
| hil-miR-6055-5p | -0.137 | 0.939 | 0.019 | 0.992 |
| hil-miR-928-5p | -0.135 | NA | -1.435 | NA |
| hil-miR-9878-3p | 6.818 | 1.45E-05 | 6.973 | 2.19E-05 |
| hil-miR-9893-5p | -0.136 | NA | 0.019 | NA |
| hil-miR-263b-3p | -0.136 | NA | 0.019 | NA |
| hil-miR-2744 | -0.135 | NA | 0.020 | NA |
| hil-miR-2774a | -0.137 | 0.939 | 0.018 | 0.992 |
| hil-miR-2775b | -0.137 | 0.939 | 0.018 | 0.992 |
| hil-miR-2780a-5p | -0.136 | NA | -1.530 | NA |
| hil-miR-279d-5p | -0.135 | 0.939 | 0.020 | 0.992 |
| hil-miR-2801 | -0.135 | 0.939 | 0.020 | 0.992 |
| hil-miR-2807a | 1.414 | NA | 1.569 | NA |
| hil-miR-2838 | -0.135 | NA | 0.020 | NA |
| hil-miR-2856-3p | 1.591 | NA | 1.746 | NA |
| hil-miR-2999 | -0.137 | 0.939 | 0.019 | 0.992 |
| hil-miR-308-5p | -0.137 | 0.939 | 0.018 | 0.992 |
| hil-miR-3212 | -1.193 | NA | 0.019 | NA |
| hil-miR-3213-3p | -0.135 | NA | 0.020 | NA |
| hil-miR-3213-5p | -0.135 | NA | 0.020 | NA |
| hil-miR-3227 | -1.864 | 0.332 | 2.581 | 0.295 |
| hil-miR-3228 | -0.135 | NA | 0.020 | NA |
| hil-miR-3238 | -4.485 | 0.003 | 0.020 | 0.992 |
| hil-miR-3264 | -0.135 | 0.939 | -2.191 | 0.477 |
| hil-miR-3265 | -0.136 | NA | 0.019 | NA |
| hil-miR-3276 | -6.552 | 1.34E-05 | 0.020 | 0.992 |
| hil-miR-3279 | 2.142 | NA | 2.297 | NA |
| hil-miR-3296-5p | 3.210 | 0.070 | 3.365 | 0.093 |
| hil-miR-3323 | -0.136 | NA | 0.019 | NA |
| hil-miR-3338-5p | 0.754 | NA | 0.909 | NA |
| hil-miR-3375-3p | -0.135 | NA | 0.020 | NA |
| hil-miR-3381-3p | 2.968 | 0.104 | 3.124 | 0.136 |
| hil-miR-140-5p | -0.137 | 0.939 | 0.019 | 0.992 |
| hil-miR-144-5p | 11.576 | 2.99E-12 | -0.325 | 0.992 |
| hil-miR-146c-3p | -0.135 | NA | -1.321 | NA |
| hil-miR-181b-3p | -0.136 | 0.939 | 0.019 | 0.992 |
| hil-miR-223-3p | 0.984 | NA | 0.192 | NA |
| hil-miR-490-5p | -2.270 | NA | 0.020 | NA |
| hil-bantam-3p | 4.527 | 1.43E-08 | -0.159 | 0.992 |
| hil-let-7-5p | 10.656 | 2.76E-17 | -0.096 | 0.992 |
| hil-miR-1000-5p | 8.292 | 4.40E-11 | -0.015 | 0.992 |
| hil-miR-1002-5p | 3.254 | 0.001 | -1.016 | 0.613 |
| hil-miR-100-5p | 12.669 | 1.83E-26 | -0.351 | 0.992 |
| hil-miR-10-5p | 1.269 | 0.194 | -2.240 | 0.014 |
| hil-miR-124-3p | 6.838 | 2.64E-05 | 0.523 | 0.992 |
| hil-miR-125-5p | 9.470 | 3.35E-16 | -0.224 | 0.992 |
| hil-miR-12-5p | 4.739 | 1.76E-06 | -1.203 | 0.539 |
| hil-miR-133-3p | 7.110 | 3.18E-09 | 0.210 | 0.992 |
| hil-miR-137-3p | -0.135 | NA | -2.832 | NA |
| hil-miR-1-3p | 6.219 | 8.35E-12 | -0.370 | 0.992 |
| hil-miR-14-3p | 5.295 | 1.01E-08 | -0.481 | 0.992 |
| hil-miR-14-5p | -0.135 | 0.939 | -4.095 | 0.017 |
| hil-miR-184-3p | 4.659 | 4.05E-08 | 0.099 | 0.992 |
| hil-miR-190-5p | 3.086 | 2.82E-04 | -0.290 | 0.992 |
| hil-miR-193-3p | 6.034 | 0.001 | -1.599 | 0.659 |
| hil-miR-210-3p | 11.345 | 6.78E-20 | -0.143 | 0.992 |
| hil-miR-2280-3p | -0.136 | NA | -1.622 | NA |
| hil-miR-2492-5p | -0.136 | NA | -1.314 | NA |
| hil-miR-2499-3p | 1.252 | 0.686 | 2.691 | 0.214 |
| hil-miR-252-5p | 5.002 | 1.45E-05 | -0.414 | 0.992 |
| hil-miR-263a-5p | 5.011 | 5.08E-09 | -0.535 | 0.992 |
| hil-miR-263b-5p | 5.478 | 2.15E-05 | -0.652 | 0.992 |
| hil-miR-274-5p | 10.117 | 9.86E-17 | -0.169 | 0.992 |
| hil-miR-275-3p | 0.704 | 0.939 | -1.085 | 0.992 |
| hil-miR-275-5p | 9.764 | 7.37E-07 | 2.152 | 0.539 |
| hil-miR-276a-3p | 6.404 | 3.12E-10 | -0.051 | 0.992 |
| hil-miR-277-3p | 9.540 | 1.60E-21 | 0.035 | 0.992 |
| hil-miR-278-3p | 2.597 | 0.010 | 0.119 | 0.992 |
| hil-miR-279-3p | 4.731 | 1.55E-07 | -0.830 | 0.827 |
| hil-miR-281-2-5p | 4.375 | 5.52E-05 | 0.062 | 0.992 |
| hil-miR-282-5p | 1.060 | 0.757 | -0.344 | 0.992 |
| hil-miR-283-5p | 1.701 | 0.061 | -0.781 | 0.793 |
| hil-miR-285-3p | 2.785 | 0.016 | -0.207 | 0.992 |
| hil-miR-2a-3p | 5.166 | 7.00E-07 | -0.300 | 0.992 |
| hil-miR-304-5p | 4.942 | 1.78E-08 | -1.421 | 0.226 |
| hil-miR-305-5p | 1.741 | 0.144 | -0.879 | 0.889 |
| hil-miR-307a-3p | 7.026 | 1.15E-07 | -0.072 | 0.992 |
| hil-miR-311-5p | -0.138 | 0.939 | 0.017 | 0.992 |
| hil-miR-314-5p | -0.136 | NA | -1.707 | NA |
| hil-miR-315-5p | 4.811 | 3.90E-05 | -0.624 | 0.992 |
| hil-miR-316-5p | 2.565 | 0.011 | -0.419 | 0.992 |
| hil-miR-317-3p | -8.325 | 8.35E-06 | -10.383 | 3.08E-08 |
| hil-miR-317-5p | 8.849 | 6.57E-08 | 5.319 | 0.003 |
| hil-miR-31b-5p | 4.752 | 6.10E-08 | 0.300 | 0.992 |
| hil-miR-33-5p | 3.652 | 4.02E-05 | -0.751 | 0.917 |
| hil-miR-3-3p | 9.553 | 2.97E-11 | 2.218 | 0.152 |
| hil-miR-34-3p | -0.135 | 0.939 | 0.020 | 0.992 |
| hil-miR-34-5p | 3.016 | 0.073 | -1.376 | 0.827 |
| hil-miR-375-3p | 1.977 | 0.013 | 0.020 | 0.992 |
| hil-miR-4-3p | 2.374 | 0.217 | -3.322 | 0.070 |
| hil-miR-4982-3p | -0.136 | 0.939 | 0.019 | 0.992 |
| hil-miR-4986-3p | -0.136 | NA | -1.924 | NA |
| hil-miR-5-5p | 6.380 | 6.23E-07 | 1.287 | 0.413 |
| hil-miR-6-2-5p | -0.134 | 0.939 | 0.021 | 0.992 |
| hil-miR-7-5p | 6.764 | 3.80E-11 | -1.599 | 0.178 |
| hil-miR-8-3p | 3.443 | 0.003 | -0.868 | 0.992 |
| hil-miR-87-3p | 4.443 | 0.001 | -0.319 | 0.992 |
| hil-miR-927-5p | 8.666 | 2.36E-18 | 0.096 | 0.992 |
| hil-miR-929-3p | 2.290 | NA | 2.445 | NA |
| hil-miR-929-5p | 4.903 | 0.004 | 0.039 | 0.992 |
| hil-miR-92a-3p | 4.589 | 2.20E-05 | -1.686 | 0.292 |
| hil-miR-932-5p | 7.463 | 3.07E-07 | 0.573 | 0.992 |
| hil-miR-9378-5p | -0.133 | 0.939 | -2.199 | 0.477 |
| hil-miR-9385-5p | -1.686 | NA | 0.019 | NA |
| hil-miR-956-3p | -0.135 | 0.939 | 0.020 | 0.992 |
| hil-miR-957-3p | 9.690 | 5.26E-16 | 0.459 | 0.992 |
| hil-miR-958-3p | -0.269 | 0.939 | -0.896 | 0.992 |
| hil-miR-961-5p | -0.139 | 0.939 | -2.026 | 0.413 |
| hil-miR-965-3p | 6.043 | 3.09E-05 | -0.589 | 0.992 |
| hil-miR-970-3p | 6.604 | 1.79E-10 | -0.389 | 0.992 |
| hil-miR-971-3p | 4.354 | 0.003 | 0.406 | 0.992 |
| hil-miR-976-5p | -1.482 | NA | 0.020 | NA |
| hil-miR-977-3p | 1.191 | NA | -0.319 | NA |
| hil-miR-980-3p | 5.689 | 1.04E-06 | -0.335 | 0.992 |
| hil-miR-981-3p | 7.156 | 7.35E-07 | -0.426 | 0.992 |
| hil-miR-988-3p | 5.216 | 0.001 | -1.666 | 0.381 |
| hil-miR-988-5p | 2.733 | 0.102 | 2.888 | 0.131 |
| hil-miR-993-3p | 4.986 | 0.001 | 0.491 | 0.992 |
| hil-miR-993-5p | -0.135 | 0.939 | -2.405 | 0.364 |
| hil-miR-999-3p | 6.284 | 9.69E-10 | -0.022 | 0.992 |
| hil-miR-9a-3p | 5.494 | 0.005 | -1.035 | 0.992 |
| hil-miR-9a-5p | 2.410 | 0.011 | -0.573 | 0.992 |
| hil-miR-iab-4-5p | 4.607 | 0.002 | -2.985 | 0.029 |
| hil-miR-iab-8-5p | 7.061 | 1.74E-08 | 7.216 | 1.36E-08 |
| hil-miR-193-5p | -1.786 | NA | 0.020 | NA |
| hil-miR-2548-3p | -3.760 | 0.019 | -1.797 | 0.588 |
| hil-miR-311-5p | -8.238 | 1.39E-07 | 0.020 | 0.992 |
| hil-miR-iab-8-3p | -0.135 | NA | 0.020 | NA |
| hil-miR-973-5p | 6.160 | 6.06E-05 | 6.315 | 9.61E-05 |
| hil-miR-2498b-5p | -0.135 | 0.939 | 0.020 | 0.992 |
| hil-miR-2765 | 7.016 | 5.97E-07 | -2.881 | 0.024 |
| hil-miR-2767 | -0.136 | NA | 0.019 | NA |
| hil-miR-2796 | 6.929 | 6.65E-07 | 0.500 | 0.992 |
| hil-miR-2a | 1.736 | 0.138 | -0.045 | 0.992 |
| hil-miR-3338 | -0.135 | 0.939 | -2.522 | 0.295 |
| hil-miR-6098 | 5.892 | 8.11E-05 | -0.670 | 0.992 |
| hil-miR-750 | -0.135 | NA | -2.800 | NA |
| hil-miR-219-3p | -0.135 | NA | -2.121 | NA |
| hil-miR-3805a-5p | -0.136 | 0.939 | -5.138 | 0.001 |
| hil-miR-3839-3p | -0.135 | 0.939 | 0.021 | 0.992 |
| hil-miR-3844-3p | -0.135 | NA | 0.020 | NA |
| hil-miR-3851o-2-5p | -0.135 | NA | 0.020 | NA |
| hil-miR-3859-5p | 4.251 | 0.006 | 4.406 | 0.008 |
| hil-miR-3861-5p | -0.135 | NA | 0.020 | NA |
| hil-miR-3862-3p | -0.137 | 0.939 | 0.018 | 0.992 |
| hil-miR-3884-5p | -3.642 | 0.031 | 0.020 | 0.992 |
| hil-miR-6011-5p | -0.136 | 0.939 | -6.527 | 8.27E-05 |
