# Supplementary material for: microRNA profile of Hermetia illucens (black soldier fly) and its implications on mass rearing
Source: PLoS One. 2022 Mar 17;17(3):e0265492. doi: 10.1371/journal.pone.0265492 (PMC8929568; doi:10.1371/journal.pone.0265492)
Supplement: Supplementary file 2 [file pone.0265492.s001.docx]

**S1 Table. Conserved *H. illucens* miRNA identified in other arthropod species.**
miRNA provisionally named using *hil* for species and number taken from miRNA identified in other arthropod species. Identical locations collapsed and those with multiple locations included below the bold. If no other location is listed, all miRNA were found on the same location.
*Life stage abbreviations: E=Egg; L=Larva; P=Pupa; F=Female; M=Male

| miRNA *H.illucens* | Life Stage* | Length (nt) | Mature Sequence | Chromosomal (CHR) Location | | | |
|---|---|---|---|---|---|---|---|
| **hil-miR-11927** | **F** | **18** | **aaaaagaucgucaucuga** | **5** | 100529647 | 100529731 | - |
| **hil-miR-10361b-3p** | **L; P** | **18** | **aaaauggucgguacaaug** | **3** | 100641948 | 100642016 | - |
| **hil-miR-5-5p** | **F; M** | **23** | **aaaggaaccaucaaugugagaug** | **3** | 74335108 | 74335168 | - |
| **hil-miR-283-5p** | **E;L;P,F,M** | **23** | **aaauaucaguugguaaauucuggg** | **5** | 78708512 | 78708576 | - |
| **hil-miR-929-5p** | **P;F;M** | **21** | **aaauugacucuaguagggagu** | **3** | 43096789 | 43096847 | + |
| **hil-miR-100-5p** | **E;P;F;M** | **22** | **aacccguagauccgaacuugug** | **4** | 72410015 | 7241007 | - |
| **hil-miR-6055-5p** | **E** | **23** | **aagacgaagggcgcgccggacgg** | **4** | 55779648 | 55779721 | - |
| **hil-miR-281-2-5p** | **E;L;P;F;M** | **22** | **aagagagcuauccgucgacagu** | **1** | 39007412 | 39007470 | + |
| **hil-miR-3227** | **L;M** | **21** | **aagcaauugauugucggcauc** | **6** | 99897234 | 99897278 | - |
| **hil-miR-6042-3p** | **E;L;P;F;M** | **22** | **aaggaacgaucguugugauauc** | **4** | 124627187 | 124627247 | + |
| **hil-miR-2a-3p** | **L;F;M** | **19** | **aaucacaggaguauacugu** | **4** | 113107875 | 113107929 | + |
| **hil-miR-3805a-5p** | **E;F** | **22** | **aaucacaucagcucuuuucuau** | **4** | 124626532 | 124626604 | + |
| **hil-miR-279d-5p** | **P** | **18** | **aaugagugggggugaaguc** | **4** | 5324583 | 5324640 | - |
| **hil-miR-263a-5p** | **E;L;P;F;M** | **24** | **aauggcacuggaagaauucacggg** | **1** | 138404014 | 138404086 | + |
| **hil-miR-10361b-5p** | **F** | **20** | **acacgauauagggacugcca** | **4** | 136417895 | 136417946 | + |
| **hil-miR-3859-5p** | **P;M** | **18** | **accccacaccugacacca** | **6** | 91542431 | 91542490 | - |
| **hil-miR-10-5p** | **E;L;P;F;M** | **22** | **acccuguagauccgaauuuguu** | **3** | 2075187 | 2075245 | + |
| **hil-miR-iab-4-5p** | **E;P;F;M** | **22** | **acguauacugaauguauccuga** | **3** | 389420 | 389478 | - |
| **hil-miR-2a-5p** | **E** | **23** | **acucucaaagugguugugaaaug** | **6** | 28456131 | 28456191 | + |
| **hil-miR-190-5p** | **E;L;P;F;M** | **24** | **agauauguuugauauucuugguug** | **2** | 147421568 | 147421629 | + |
| **hil-miR-6011-5p** | **E;P;F** | **25** | **agaugaugcaacuuugaauggugag** | **3;7** | | | |
| | | | agaugaugcaacuuugaauggugag | 3 | 90462080 | 90462124 | + |
| | | | agaugaugcaacuuugaauggugag | 3 | 90462101 | 90462150 | + |
| | | | agaugaugcaacuuugaauggugag | 7 | 7194175 | 7194224 | + |
| **hil-miR-11903b** | **F** | **25** | **agccauauuaucguucgagcugaca** | **1** | 97288015 | 97288101 | + |
| **hil-miR-311-5p** | **P** | **18** | **aggacggugucguagacc** | **2** | 8609521 | 8609570 | - |
| **hil-miR-iab8-3p** | **E** | **23** | **aggauacauucaguauacguaca** | **3** | 389421 | 389481 | + |
| **hil-miR-973-5p** | **P;M** | **18** | **aggguuggcucugaagau** | **5** | 13228815 | 13228862 | + |
| **hil-miR-2775b** | **P** | **18** | **aguaggacauugcuaggc** | **1;2** | | | |
| | | | aguaggacauugcuaggc | 1 | 168226734 | 168226771 | - |
| | | | aguaggacauugcuaggc | 2 | 54877373 | 54877411 | - |
| **hil-miR-223-3p** | **F;M** | **22** | **agucaguuguucuuuucuucaa** | **3** | 60062104 | 60062165 | - |

| miRNA *H.illucens* | Life Stage* | Length (nt) | Mature Sequence | Chromosomal (CHR) Location | | | |
|---|---|---|---|---|---|---|---|
| **hil-miR-9378-5p** | **E;F** | **19** | **aguggaguugucacuuuuc** | **2** | 46865742 | 46865784 | + |
| **hil-miR-4-3p** | **E;L;P;F;M** | **23** | **auaaagcuagauuaccaaagcau** | **1;4** | | | |
| | | | auaaagcuagauuaccaaagcau | 1 | 158777862 | 158777923 | + |
| | | | auaaagcuagaucaccaaugca | 4 | 124627018 | 124627078 | + |
| **hil-miR-314-5p** | **F** | **18** | **auaacuuggcgauuggac** | **2** | 138688743 | 138688804 | - |
| **hil-miR-9893-5p** | **E** | **25** | **auaugaucgagucaagcagugccgg** | **3** | 109202652 | 109202717 | - |
| **hil-3381-3p** | **M** | **25** | **auauucauaugaccgucguccgggu** | **3** | 152519090 | 152519158 | + |
| **hil-miR-1000-5p** | **E;P;F;M** | **22** | **auauuguccugucacagcagua** | **6** | 1819714 | 1819771 | + |
| **hil-miR-181b-3p** | **P** | **18** | **aucacugaaagucggccu** | **5** | 75841530 | 75841579 | + |
| **hil-miR-3752-3p** | **L** | **19** | **auggauaaccgcugaaggc** | **3** | 141380225 | 141380273 | - |
| **hil-miR-305-5p** | **E;L;P;F;M** | **24** | **auuguacuucaucaggugcucugg** | **1** | 190924436 | 190924495 | - |
| **hil-miR-2498b-5p** | **P** | **20** | **caaagucugaugugaaaucc** | **2** | 185673737 | 185673826 | + |
| **hil-miR-304-5p** | **E;L;P;F;M** | **23** | **caaucucaaauuguuauuguggu** | **5** | 78702696 | 78702759 | - |
| **hil-miR-3213-5p** | **P** | **23** | **cacuacuugaucgcgacacucgg** | **3** | 100784348 | 100784398 | + |
| **hil-miR-10373-3p** | **F** | **23** | **cagagaaagaugucaggcugguc** | **1** | 22883734 | 22883821 | - |
| **hil-miR-3228** | **P** | **19** | **cagaucaacuguaguuaag** | **2** | 14166820 | 14166910 | - |
| **hil-miR-34-3p** | **E** | **23** | **cagccacuaucccucccugccgcc** | **3** | 44268799 | 44268861 | - |
| **hil-miR-311-5p** | **L;P** | **21** | **cagcugggagagcaucuggcu** | **4** | 66232631 | 66232688 | + |
| **hil-miR-2780a-5p** | **F** | **18** | **cagggugugguugugggc** | **3** | 151344004 | 151344062 | + |
| **hil-miR-2a-3p** | **L;P;F;M** | **22** | **caucacagucugaguucuugcu** | **6** | 6765948 | 6766012 | - |
| **hil-miR-490-5p** | **L** | **24** | **ccauggauucaguacguagcggac** | **5** | 39325217 | 39325308 | - |
| **hil-miR-2856-3p** | **M** | **20** | **ccauucgaggacagucuggc** | **2** | 92165123 | 92165191 | + |
| **hil-miR-988-3p** | **P;F;M** | **22** | **ccccuuguugcaaaccccacgu** | **1** | 102645320 | 102645378 | - |
| **hil-miR-278-5p** | **E** | **23** | **ccggacgagggucucagcgacc** | **4** | 137680214 | 137680273 | + |
| **hil-miR-977-3p** | **F;M** | **22** | **cgagauauucaaaucguccagu** | **6** | 37625503 | 37625569 | - |
| **hil-miR-3338-5p** | **M** | **22** | **cgcaaacagggcggauuaggca** | **7** | 12253510 | 12253560 | - |
| **hil-miR-275-5p** | **F;M** | **23** | **cgcgcuaagcaggcgccagggcu** | **1** | 190924617 | 190924673 | - |
| **hil-miR-3375-5p** | **P** | **20** | **cgcuaucuggguucagaacg** | **3** | 107907703 | 107907752 | - |
| **hil-miR-311-5p** | **E** | **22** | **cggacgguuguugggguugcagc** | **2** | 20179181 | 20179240 | + |
| **hil-miR-14-5p** | **E;F** | **18** | **cggagcgaggcuuggcca** | **4** | 59552727 | 59552773 | + |
| **hil-miR-6-2-5p** | **E** | **23** | **cgggaacucuugcugugacugcu** | **6** | 6765948 | 6766012 | - |
| **hil-miR-1-371-5p** | **M** | **22** | **cguuugauugauucccugaguc** | **4** | 120681598 | 120681663 | + |

| miRNA *H.illucens* | Life Stage* | Length (nt) | Mature Sequence | Chromosomal (CHR) Location | | | |
|---|---|---|---|---|---|---|---|
| **hil-miR-252-5p** | **E;L;P;F;M** | **22** | **cuaaguacuagugccgcaggag** | **5** | 52093567 | 52093622 | - |
| **hil-miR-6098** | **P;F;M** | **22** | **cuagugaucagucgacaucgau** | **1** | 57033124 | 57033184 | + |
| **hil-miR-3862-3p** | **P** | **18** | **cucaggcgguuagagcgc** | **5** | 32965086 | 32965130 | + |
| **hil-miR-929-3p** | **M** | **21** | **cucccuaacggagucagauug** | **3** | 43096789 | 43096847 | + |
| **hil-miR-3338** | **F** | **23** | **cuguacuugaucgcggcacucgg** | **5** | 110263129 | 110263194 | - |
| **hil-miR-961-5p** | **E;F** | **25** | **cuugaucaguaggcuuucgguuuuu** | **1** | 183158507 | 183158596 | - |
| **hil-miR-263b-5p** | **E;P;F;M** | **23** | **cuuggcacugggagaauucacag** | **2** | 145800145 | 145800204 | + |
| **hil-miR-2548-3p** | **F** | **19** | **cuuggugugaaagguagacg** | **2** | 172424800 | 172424867 | + |
| **hil-miR-210-3p** | **P;F;M** | **21** | **cuugugcgugugacagcggcu** | **5** | 70458922 | 70458979 | + |
| **hil-miR-993-3p** | **E;P;F;M** | **23** | **gaagcucgucucuacagguaucu** | **3** | 29290170 | 29290254 | - |
| **hil-miR-3323** | **E** | **25** | **gaauacgauccuacggcggagcagc** | **3** | 96780080 | 96780148 | - |
| **hil-miR-3238** | **L** | **20** | **gaaugaauaucgugcaggau** | **4** | 8579804 | 8579865 | + |
| **hil-miR-11913** | **P** | **19** | **gauguuggugauccugugu** | **4** | 128395253 | 128395306 | + |
| **hil-miR-2280-3p** | **F** | **20** | **gauuuacucugaacccgcga** | **4** | 140003993 | 140004069 | + |
| **hil-miR-10357-5p** | **P** | **18** | **gcagaaccuuaccuggcc** | **1** | 180215809 | 180215844 | + |
| **hil-miR-3884-5p** | **L** | **18** | **gcugaagccggucccaag** | **2** | 70251684 | 70251742 | - |
| **hil-miR-3777-3p** | **P** | **19** | **gggaaacagaaccugaaac** | **4** | 43209498 | 43209567 | - |
| **hil-miR-9878-3p** | **M** | **19** | **ggggauguagcucaguggu** | **3** | 102480797 | 102480859 | + |
| **hil-miR-309b-5p** | **F** | **23** | **ggucaaaccccguucaguuggua** | **4** | 124627494 | 124627553 | + |
| **hil-miR-12418-3p** | **P** | **19** | **gguucgauuccgguacucg** | **2** | 100712477 | 100712526 | + |
| **hil-miR-2796** | **E;P;F;M** | **23** | **guaggccggcggaaacuacuugc** | **6** | 54741226 | 54741285 | + |
| **hil-miR-3212** | **L** | **22** | **gucacugugucgucgucgggga** | **6** | 90271124 | 90271178 | - |
| **hil-miR-2774a** | **P** | **18** | **gucggugguuuuagcggu** | **6** | 87657009 | 87657073 | - |
| **hil-miR-2999** | **E** | **18** | **gugcgacggcuucuuuuu** | **1** | 44402081 | 44402133 | - |
| **hil-miR-988-5p** | **E;M** | **22** | **gugugauuuguggcaaugggau** | **1** | 102645320 | 102645378 | - |
| **hil-miR-9a-3p** | **E;P;F;M** | **22** | **uaaagcuagauuaccaaagcau** | **1** | 158777862 | 158777923 | + |
| **hil-miR-277-3p** | **E;L;P;F;M** | **23** | **uaaaugcacuaucugguacgaca** | **3** | 44278531 | 44278596 | - |
| **hil-miR-965-3p** | **E;L;P;F;M** | **22** | **uaagcguauagcuuuuccccuu** | **1** | 137738280 | 137738342 | - |
| **hil-miR-124-3p** | **E;P;F;M** | **21** | **uaaggcacgcggugaaugcca** | **3** | 124681385 | 124681442 | + |
| **hil-miR-2767** | **E** | **25** | **uaaguaaacucacggcgcagcgagc** | **1** | 221172208 | 221172261 | + |

| miRNA *H.illucens* | Life Stage* | Length (nt) | Mature Sequence | Chromosomal (CHR) Location | | | |
|---|---|---|---|---|---|---|---|
| **hil-miR-970-3p** | **E;L;P;F;M** | **21** | **ucauaagacacacgcggcuau** | **5** | 90556233 | 90556296 | + |
| **hil-miR-4986-3p** | **F** | **21** | **ucaucggacgcugcuucaccu** | **1** | 131419780 | 131419829 | - |
| **hil-miR-125-5p** | **P;F;M** | **22** | **ucccugagacccuaacuuguga** | **4** | 72383122 | 72383181 | - |
| **hil-miR-278-3p** | **L;P;F;M** | **22** | **ucggugggacuuucguccguuu** | **4** | 137680214 | 137680272 | + |
| **hil-miR-2a-5p** | **E;L** | **23** | **ucucucaaagugguugugaaaug** | **6** | 28447811 | 28447876 | + |
| **hil-miR-998-5p** | **E;L;P** | **22** | **ucugaacucucaugggccugca** | **6** | 6758949 | 6759006 | - |
| **hil-miR-3265** | **E** | **23** | **ucugagaggccagcuugggcgag** | **4** | 63355567 | 63355613 | - |
| **hil-miR-38390-3p** | **E** | **24** | **ucuuguuggaaauguacuuggagc** | **1;4** | | | |
| | | | ucuuguuggaaauguacuuggagc | 1 | 6048991 | 6049061 | + |
| | | | ucuuguuggaaauguacuuggagc | 4 | 80857861 | 80857945 | + |
| **hil-miR-9a-5p** | **E;L;P;F;M** | **22** | **ucuuugguauucuagcuguaga** | **1;2** | | | |
| | | | ucuuugguauucuagcuguaga | 1 | 158760631 | 158760693 | + |
| | | | ucuuuggugauuaagcucugugg | 1 | 158778316 | 158778379 | + |
| | | | ucuuugguuaucuagcuguauga | 2 | 61885951 | 61886011 | - |
| **hil-miR-3279** | **M** | **22** | **ucuuuucaacaacugaccuuca** | **5** | 37098245 | 37098303 | - |
| **hil-miR-957-3p** | **E;P;F;M** | **22** | **ugaaaccguccaaaacugaggc** | **2** | 277009 | 277075 | + |
| **hil-miR-317-3p** | **E;L;P;F** | **25** | **ugaacacagcugguguaucucagu** | **3** | 44334763 | 44334824 | - |
| **hil-miR-279-3p** | **E;L;P;F;M** | **22** | **ugacuagauuugcacucauccg** | **3;4** | | | |
| | | | ugacuagauuugcacucauccg | 3 | 162862403 | 162862464 | + |
| | | | ugacuagauccacacucauuaa | 3 | 71661045 | 71661110 | + |
| | | | ugacuagauaucaugcucgucu | 3 | 71685196 | 71685263 | + |
| | | | ugacuagaccgaacacucgucagc | 4 | 124626873 | 12462693 | + |
| **hil-bantam-3p** | **E;L;P;F;M** | **23** | **ugagaucauuuugaaagcugauu** | **2** | 172657695 | 172657755 | - |
| **hil-miR-958-3p** | **L;P;F;M** | **21** | **ugagauucuucuauucuacuu** | **7** | 9454370 | 9454430 | - |
| **hil-let-7-5p** | **P;F;M** | **21** | **ugagguaguagguuguauagu** | **4** | 72384272 | 72384330 | - |
| **hil-miR-12-5p** | **E;L;P;F;M** | **23** | **ugaguauuauauuagguacuggu** | **5** | 78702132 | 78702198 | - |
| **hil-miR-144-5p** | **P;F;M** | **23** | **ugauaucauuuuguaagcugauu** | **1** | 80122407 | 80122459 | - |
| **hil-miR-3213-3p** | **E** | **23** | **ugauaugaaauuugggagaaagg** | **4** | 150288034 | 150288086 | - |
| **hil-miR-4982-3p** | **E** | **24** | **ugcuggaacugcaggucgguugac** | **1** | 97399443 | 97399519 | - |
| **hil-miR-7-5p** | **E;L;P;F;M** | **24** | **uggaagacuagugauuuuguuguu** | **6** | 461937 | 461998 | + |
| **hil-miR-1-3p** | **E;L;P;F;M** | **22** | **uggaauguaaagaaguauggag** | **3** | 176783749 | 176783810 | - |
| **hil-miR-184-3p** | **E;L;P;F;M** | **22** | **uggacggagaacugauaagggc** | **1** | 43471172 | 43471232 | + |
| **hil-miR-219-3p** | **F** | **23** | **uggacugugacucaacugacgcc** | **7** | 769423 | 769477 | + |
| **hil-miR-31b-5p** | **E;L;P;F;M** | **23** | **uggcaagaugucggcauagcuga** | **1** | 47357143 | 47357205 | + |
| **hil-miR-34-5p** | **E;L;P;F;M** | **24** | **uggcaguguggguuagcugguugug** | **3** | 44268799 | 44268861 | - |

| miRNA *H.illucens* | Life Stage* | Length (nt) | Mature Sequence | Chromosomal (CHR) Location | | | |
|---|---|---|---|---|---|---|---|
| **hil-miR-317-5p** | **L;F;M** | **22** | **ugggauacacucugugcucgcu** | **3** | 44334763 | 44334824 | - |
| **hil-miR-2765** | **P;F;M** | **23** | **ugguaacuccaccaccguuggcg** | **6** | 8129158 | 8129216 | + |
| **hil-miR-316-5p** | **E;L;P;F;M** | **22** | **ugucuuuuuccgcuuacugccg** | **2** | 30659317 | 30659377 | - |
| **hil-miR-308-5p** | **P** | **20** | **ugugguauccgagcuggacu** | **4** | 91412364 | 91412417 | + |
| **hil-miR-263a-3p** | **M** | **22** | **uguguucuuuguacuauggacu** | **3** | 133966825 | 133966911 | - |
| **hil-miR-999-3p** | **E;L;P;F;M** | **22** | **uguuaacuguaagacugugucu** | **3** | 22030039 | 22030098 | - |
| **hil-miR-3851o-2-5** | **E** | **23** | **uguuggcguugauuugauguggu** | **7** | 10614134 | 10614188 | + |
| **hil-miR-193-5p** | **L** | **18** | **uguugggaaguuggugau** | **4** | 73469962 | 73470002 | - |
| **hil-miR-1002-5p** | **E;L;F;M** | **23** | **uuaaguaguaaauaaguagguga** | **3** | | | |
| | | | uuaaguaguaaauaaguaggug | 3 | 180202834 | 180202894 | + |
| | | | uuaaguaguaaauaaguagguga | 3 | 180203029 | 180203089 | + |
| **hil-miR-iab-8-5p** | **M** | **22** | **uuacguauacugaagguauacc** | **3** | 389421 | 389481 | + |
| **hil-miR-2807a** | **M** | **22** | **uuccagcuaaugaccucugaug** | **3** | 164949999 | 164950080 | + |
| **hil-miR-146c-3p** | **F** | **24** | **uuccaugguaaacucuugaggaga** | **7** | 1516677 | 1516765 | - |
| **hil-miR-2940-3p** | **F** | **23** | **uucgacaggaauucugauggacu** | **4** | 148574138 | 148574203 | - |
| **hil-miR-3724-3p** | **F** | **21** | **uucgugguauuuguuggcugc** | **1** | 104888228 | 104888287 | + |
| **hil-miR-981-3p** | **E;P;F;M** | **22** | **uucguugucgacgaaaccugca** | **5** | 58835647 | 58835708 | - |
| **hil-miR-263b-3p** | **P** | **19** | **uugaauuugauacugggug** | **1** | 50551838 | 50551912 | - |
| **hil-miR-87-3p** | **P;F;M** | **23** | **uugagcaaaauuucaggugugug** | **1** | 162813456 | 162813518 | + |
| **hil-miR-33-5p** | **E;L;P;F;M** | **21** | **uugcauuguagucgcauugua** | **5** | 54162908 | 54162968 | + |
| **hil-miR-2499-3p** | **E;L;M** | **22** | **uugcggaucuauggacgucucu** | **1** | 161572119 | 161572180 | + |
| **hil-miR-2801** | **E** | **22** | **uuggaaucgauugguaacuggc** | **2** | 98304661 | 98304709 | + |
| **hil-miR-3861-5p** | **P** | **23** | **uuggaauuucucugagacauuuu** | **2** | 41199313 | 41199393 | + |
| **hil-miR-928-5p** | **F** | **25** | **uuggcuguguugcuggagcuggcgu** | **4** | 35515572 | 35515665 | + |
| **hil-miR-2492-5p** | **F** | **23** | **uugggaucaggccauuuugcugu** | **4** | 76411949 | 76412008 | - |
| **hil-miR-133-3p** | **E;L;P;F;M** | **22** | **uugguccccuucaaccagcugu** | **3** | 176248355 | 176248417 | - |
| **hil-miR-971-3p** | **P;F;M** | **21** | **uugguguuauaucuuacagug** | **4** | 39613879 | 39613936 | + |
| **hil-miR-11894a** | **F** | **22** | **uuuaccauuaucguugcggccc** | **6** | 26336958 | 26337006 | + |
| **hil-miR-927-5p** | **L;P;F;M** | **22** | **uuuagaauuccuacgcuuuacc** | **5** | 3001374 | 3001432 | + |
| **hil-miR-956-3p** | **E;P** | **20** | **uuucgagaccacuacaaacu** | **2** | 55097617 | 55097700 | - |
| **hil-miR-2744** | **P** | **18** | **uuugagaguuugauccug** | **2** | 3655097 | 3655150 | - |
| **hil-miR-976-5p** | **L** | **19** | **uuuggugauuauggcggag** | **3** | 75118931 | 75118996 | - |

| miRNA *H.illucens* | Life Stage* | Length (nt) | Mature Sequence | Chromosomal (CHR) Location | | | |
|---|---|---|---|---|---|---|---|
| **hil-miR-2548-3p** | **L** | **19** | **uuugguguuguaggacugc** | **2** | 41964183 | 41964252 | + |
| **hil-miR-274-5p** | **P;F;M** | **22** | **uuugugaccgacacuaacgggu** | **2** | 43581222 | 43581305 | - |
| **hil-miR-3844-3p** | **E** | **23** | **uuugugugguuguggaaugagg** | **2** | 42800748 | 42800832 | - |
| **hil-miR-375-3p** | **E;L;P;F;M** | **22** | **uuuguucguuuggcuuaagugc** | **3** | 112525535 | 112525597 | + |
| **hil-miR-3296-5p** | **M** | **23** | **uuuugaguugcagagucgcuggc** | **1** | 30541241 | 30541312 | + |
| **hil-miR-315-5p** | **E;L;P;F;M** | **23** | **uuuugauuguugcucagaaagcc** | **2** | 141335340 | 141335401 | + |
