# Supplementary material for: microRNA profile of Hermetia illucens (black soldier fly) and its implications on mass rearing
Source: PLoS One. 2022 Mar 17;17(3):e0265492. doi: 10.1371/journal.pone.0265492 (PMC8929568; doi:10.1371/journal.pone.0265492)
Supplement: Supplementary file 3 [file pone.0265492.s002.docx]

S2 Table. Unique *H. illucens* miRNA not identified in any other species.
miRNA provisionally named; identical locations collapsed and those with multiple locations included below the bold. If no other location is listed, all miRNA were found on the same location.
*Life stage abbreviations: E=Egg; L=Larva; P=Pupa; F=Female; M=Male

miRNA *H.illucens*	Life Stage*	Length (nt)	Mature Sequence	Chromosomal (CHR) Location			
**hil-miR-a**	**F; M**	**22**	**aacgauuugcucccuucuccag**	**5**	97170454	97170508	-
**hil-miR-b**	**F; M**	**22**	**aaugagcccgguauucugaaca**	**1**	105897629	105897684	-
**hil-miR-f**	**E;L;P;F;M**	**22**	**agcaguguuuaugaucaggcuu**	**6**	9852651	9852712	+
**hil-miR-g**	**P;F**	**22**	**aggauugugacuggacaucgug**	**2**	174068470	174068531	-
**hil-miR-h**	**F**		**caccuaagcugucgaacaagc**	**1**	221194834	221194889	+
**hil-miR-j**	**F;P**	**21**	**ccagaacugagcgauuuaaau**	**2;6**			
			ccagaacugagcgauuuaaau	6	78783078	78783136	+
			ccagaacugagcgauuuaaau	2	73070312	73070370	+
**hil-miR-l**	**F;E**	**23**	**cccuccuugaucgcggcacucgg**	**2;3;4**			
			cccuccuuaaucgcggcacucgg	2	188148995	188149056	+
			cccuccuugaucgcggcgcucgg	4	51883729	51883807	-
			cccuccuugaucgcggcgcucgg	3	178862038	178862116	+
			cccuccuugaucgcugcacucgg	2	58573817	58573878	+
			cccugcuugaucgcggcacucgg	4	50750836	50750897	-
			cccugcuugaucgcggcacucgg	4	50757250	50757311	-
**hil-miR-m**	**E**	**22**	**cuauacgaucugcuagcuuccc**	**4**	72384272	72384330	-
**hil-miR-n**	**E;P;F;M**	**23**	**cucucucugccuuggcauucucc**	**1**	6471787	6471846	+
**hil-miR-o**	**E;L;P;F;M**	**22**	**gagagugcugucguaccugacc**	**1**	158769156	158769218	+
**hil-miR-p**	**E**	**22**	**gcagaggugaggcagcgucuga**	**7**	10424208	10424283	-
**hil-miR-r**	**E**	**23**	**ggcgaacauggaucuagugcacg**	**3**	71685196	71685262	+
**hil-miR-s**	**E**	**23**	**gguguccacugcaggccuguaug**	**3**	124681384	124681443	+
**hil-miR-t**	**F;M**	**22**	**uacuguaaugaacuuggcggaa**	**4**	149752585	149752641	-
**hil-miR-u**	**E**	**21**	**ucacuggauaaguuugucgua**	**4**	124626721	124626783	+
**hil-miR-v**	**P;F;M**	**22**	**ucagacuuugcgaucgaccuuu**	**3**	141644304	141644363	+
**hil-miR-w**	**E;L**	**22**	**ucguuaauuuggucgugacgug**	**6**	28447603	28447667	+
**hil-miR-x**	**E;F**	**23**	**ucuuuuucauuaggauccuucug**	**6**	21724849	21724909	+
**hil-miR-y**	**F;M**	**22**	**ugagccgguugaaucgcacgga**	**1**	75691513	75691575	+
**hil-miR-aa**	**P;F**	**24**	**ugcaguguauuccuguguuuuugg**	**4**	113107872	113107929	+
**hil-miR-ac**	**P**	**23**	**uucuuuucaacaacugaccuuca**	**5**	37098245	37098303	-
**hil-miR-ad**	**E;F**	**22**	**uuggucgguaguggugaggagc**	**2;7**			
			uuggucgguaguggugaggagc	7	1560975	1561018	+
			uuggucgguaguggugaggagc	2	19009895	19009959	+
**hil-miR-ae**	**P;F;M**	**22**	**uuguggaacucgaugaugcagc**	**2**	18803235	18803293	+
**hil-miR-af**	**L;M**	**25**	**uuuucggaauguuguaauaaauaua**	**7**			
			uuuucggaauguuguaauaaauaua	7	738404	738469	-
			uuuucggaauguuguaauaaauaua	7	763802	763867	+
