# Supplementary material for: microRNA profile of Hermetia illucens (black soldier fly) and its implications on mass rearing
Source: PLoS One. 2022 Mar 17;17(3):e0265492. doi: 10.1371/journal.pone.0265492 (PMC8929568; doi:10.1371/journal.pone.0265492)
Supplement: Supplementary file 4 [file pone.0265492.s003.docx]

S3 Table. Unique *H. illucens* miRNA log fold change (LFC) and P-Adjusted Value between life stages.
miRNA provisionally named; Green filled boxes represent statistically significant up regulated LFC; Red filled boxes represent statistically significant down regulated LFC.

	Egg v Larva		Larva v Pupa		Pupa v Female		Pupa v Male		Female v Male	
miRNA	LFC	P-Adjusted Value	LFC	P-Adjusted Value	LFC	P-Adjusted Value	LFC	P-Adjusted Value	LFC	P-Adjusted Value
hil-miR-a	0.539	0.795	-0.352	0.843	-0.451	0.791	-2.129	0.232	-1.678	0.359
hil-miR-aa	0.533	0.807	3.470	0.328	-1.996	0.687	3.988	0.155	5.983	0.020
hil-miR-ac	0.513	0.795	3.367	0.010	5.053	4.58E-05	3.869	0.002	-1.184	0.629
hil-miR-ad	-2.474	0.097	-0.348	0.843	-0.571	0.767	0.161	0.959	0.732	0.703
hil-miR-ae	0.514	0.807	2.690	0.594	-3.815	0.169	-1.178	0.959	2.638	0.377
hil-miR-af	10.111	6.88E-06	-9.900	7.71E-05	1.442	0.734	-13.122	1.61E-08	-14.563	9.58E-11
hil-miR-b	0.530	0.795	-0.351	0.843	-2.895	0.115	-3.079	0.064	-0.184	0.868
hil-miR-f	7.893	1.46E-10	2.777	0.010	-0.790	0.687	-1.579	0.172	-0.790	0.629
hil-miR-g	0.523	NA	0.761	0.843	0.942	0.707	1.269	0.746	0.327	0.863
hil-miR-h	0.539	0.795	-0.355	0.843	-2.147	0.250	0.171	0.959	2.318	0.176
hil-miR-j	0.526	NA	0.343	0.843	0.647	0.734	0.854	0.959	0.207	0.868
hil-miR-l	-8.254	4.03E-07	-0.349	0.843	-7.268	4.58E-05	0.166	0.959	7.434	1.88E-05
hil-miR-m	-1.835	NA	-0.349	0.843	1.713	0.668	0.154	0.959	-1.559	0.581
hil-miR-n	-3.090	0.073	1.738	0.763	-0.716	0.734	0.068	0.959	0.784	0.688
hil-miR-o	-5.743	5.12E-05	2.491	0.315	0.006	0.997	-0.579	0.959	-0.584	0.761
hil-miR-p	-6.565	3.43E-05	-0.350	0.843	1.254	0.730	0.167	0.959	-1.087	0.688
hil-miR-r	-8.903	3.49E-09	-0.350	0.843	1.353	0.708	0.168	0.959	-1.186	0.668
hil-miR-s	-2.784	0.018	-0.357	0.843	1.378	0.687	0.148	0.959	-1.230	0.629
hil-miR-t	0.538	0.795	-0.352	0.843	-0.351	0.827	-2.834	0.102	-2.484	0.104
hil-miR-u	-11.735	4.64E-24	-0.348	0.843	1.872	0.596	0.168	0.959	-1.704	0.516
hil-miR-v	0.533	0.795	1.272	0.763	-0.386	0.767	-2.354	0.044	-1.968	0.080
hil-miR-w	-10.012	2.04E-17	-2.989	0.209	1.514	0.687	0.167	0.959	-1.347	0.629
hil-miR-x	-6.230	3.41E-05	-0.350	0.843	-1.028	0.730	0.166	0.959	1.194	0.629
hil-miR-y	0.521	0.795	-0.354	0.843	-3.553	0.007	-4.602	1.70E-04	-1.049	0.359

	Egg v Pupa		Egg v Female		Egg v Male		Larva v Female		Larva v Male	
miRNA	LFC	P-Adjusted Value	LFC	P-Adjusted Value	LFC	P-Adjusted Value	LFC	P-Adjusted Value	LFC	P-Adjusted Value
hil-miR-a	-0.187	0.926	-0.639	0.707	-2.317	0.084	-0.099	0.979	-1.777	0.368
hil-miR-aa	-4.003	0.089	-5.998	0.005	-0.015	0.994	-5.466	0.020	0.518	0.809
hil-miR-ac	-3.880	0.001	1.173	0.507	-0.011	0.994	1.686	0.310	0.502	0.803
hil-miR-ad	2.822	0.063	2.251	0.068	2.983	0.036	-0.223	0.964	0.508	0.803
hil-miR-ae	-3.204	0.180	-7.019	0.001	-4.381	0.041	-6.505	0.005	-3.867	0.151
hil-miR-af	-0.211	0.926	1.230	0.707	-13.333	1.70E-09	11.342	1.58E-06	-3.222	0.264
hil-miR-b	-0.179	0.926	-3.074	0.026	-3.258	0.018	-2.544	0.119	-2.728	0.114
hil-miR-f	-10.670	9.62E-19	-11.460	1.61E-21	-12.249	2.60E-24	-3.567	1.48E-04	-4.356	0.000
hil-miR-g	-1.284	0.431	-0.342	0.816	-0.015	0.994	0.181	0.964	0.508	0.803
hil-miR-h	-0.184	0.926	-2.331	0.069	-0.013	0.994	-1.792	0.239	0.526	0.803
hil-miR-j	-0.869	0.668	-0.222	0.858	-0.015	0.994	0.304	0.964	0.511	0.803
hil-miR-l	8.603	1.30E-07	1.335	0.432	8.768	5.85E-08	-6.919	9.36E-05	0.514	0.803
hil-miR-m	2.184	0.117	3.897	0.002	2.338	0.068	2.062	0.247	0.503	0.803
hil-miR-n	1.352	0.431	0.637	0.707	1.421	0.346	-2.453	0.233	-1.669	0.596
hil-miR-o	3.253	0.039	3.258	0.030	2.674	0.068	-2.485	0.177	-3.069	0.114
hil-miR-p	6.915	1.51E-05	8.169	2.98E-07	7.082	7.70E-06	1.604	0.468	0.517	0.803
hil-miR-r	9.253	8.35E-10	10.606	7.67E-13	9.420	3.94E-10	1.703	0.427	0.517	0.803
hil-miR-s	3.141	0.008	4.519	1.57E-04	3.289	0.004	1.735	0.310	0.505	0.803
hil-miR-t	-0.187	0.926	-0.537	0.749	-3.021	0.032	0.001	0.999	-2.482	0.151
hil-miR-u	12.083	1.94E-25	13.955	1.67E-35	12.251	3.89E-26	2.220	0.233	0.516	0.803
hil-miR-v	-1.806	0.208	-2.192	0.080	-4.160	4.42E-04	-1.658	0.247	-3.627	0.009
hil-miR-w	13.000	3.90E-20	14.514	1.16E-25	13.167	7.53E-21	4.503	0.006	3.156	0.114
hil-miR-x	6.579	1.43E-05	5.552	2.19E-05	6.745	6.92E-06	-0.678	0.817	0.516	0.803
hil-miR-y	-0.167	0.926	-3.719	0.001	-4.769	1.80E-05	-3.198	0.010	-4.247	0.001
