# Supplementary material for: microRNA profile of Hermetia illucens (black soldier fly) and its implications on mass rearing
Source: PLoS One. 2022 Mar 17;17(3):e0265492. doi: 10.1371/journal.pone.0265492 (PMC8929568; doi:10.1371/journal.pone.0265492)
Supplement: Attachment — Submitted filename: ResponseLetter_PONE-D-21-33119.pdf [file pone.0265492.s006.pdf]

26 February 2022

Editor: PLoS ONE

RESPONSE: PONE-D-21-33119

Dear Editor

Please find below our responses to each of the Reviewers' comments. Our responses are in bold font.

Reviewer #1: Dear Prof. Falabella

The manuscript with ID PONE-D-21-33119 by DeRaedt and coauthors studied the microRNA profile of Hermetia illucens and its implications on mass rearing. The manuscript is interesting, tested hypothesis is well-defined and methodologies are well-described. However, I found numerous revisions that should be carefully revised by authors before the manuscript being considered for publication in PLOS ONE.

Thank you so much for your review and comments. We have gone through and made the changes and edits you have suggested.

1. Please find the attached word file with some track changes.

   We have gone through and corrected each of the track changes found in the attached document.

2. Line 57: Support with The feasibility of using yellow mealworms (Tenebrio molitor): Towards a sustainable aquafeed industry. Animals, 2021, 11(3), pp. 1–38, 811

   Line 57: Added as reference 5

3. Line 58: Support with Aquaculture, 2020, 522, 735136, Fish and Shellfish Immunology, 2021, 111, pp. 111–118

   Line 58 : added both as references 8 and 9 respectively.

South African National Bioinformatics Institute
5th Floor, Life Sciences Building, Modderdam Road, Bellville, 7535 Telephone: +27 21 959 3645 Fax: +27 21 959 2512
University of the Western Cape. Private Bag X17. Bellville 7535. South Africa.

4. All scientific names present in the references should be written italic (Please see the attached word file).

   We have gone through and made sure they are all italicized.

Reviewer #2:

In this paper authors analyzed the profile of microRNAs in all the developmental stages of Hermetia illucens. The aim was to relate them to possible functions in order to understand, in future works, the specific gene regulation to improve mass rearing techniques. I have some doubts that must be addressed, especially regarding the comparison among different life stages, it is not clear, and it seems that in many points sentences seem to be contradictory.

We have made addressed each of the issues raised below and tried to clarify our analyses between life stages.

1. KEYWORDS: I suggest to change the keywords that are already in the title

   Not sure if you are suggesting removing some of the keywords and limit them to just the ones found in our title?

2. INTRODUCTION: "Black soldier fly larvae (BSFL) contain 42% protein, 35% crude fat (7), and 8.72% chitin in prepupae (8)." This sentence is too generic, protein, lipid and even chitin content change according to the feeding substrates. I suggest to report some examples of different substrates and a range of protein, lipid and chitin content (for example https://doi.org/10.1038/s41598-019-46603-z; https://doi.org/10.1007/s12649-019-00924-2; https://doi.org/10.3390/su131810198) Authors wrote "they are an ideal candidate for large scale rearing for food sustainability and waste reduction." Although few studies are performed at industrial scale, I suggest to cite them (for example https://doi.org/10.1038/s41598-020-76571-8; https://doi.org/10.3390/ani10071243)

   Fixed Lines 61-68: "The black soldier fly (*Hermetia illucens;* BSF) is a species found worldwide and can be used as a sustainable animal and fish feed (6-9). Depending on the organic substrate the black soldier fly larvae (BSFL) are fed and the extraction process , they can contain a range of 40-60% protein, up to 47% lipid , and 3-8% chitin in prepupae (10-15). This makes them not only a good protein source, but shows potential for a variety of derived products, and has minimal environmental impacts. Due to these facts, and that they are not a

South African National Bioinformatics Institute
5th Floor, Life Sciences Building, Modderdam Road, Bellville, 7535 Telephone: +27 21 959 3645 Fax: +27 21 959 2512
University of the Western Cape. Private Bag X17. Bellville 7535. South Africa.

nuisance species, or mechanical disease vector (16, 17), they are an ideal candidate for large scale rearing for food sustainability and waste reduction (18, 19)."

We have added in references suggested, and a range for the composition of prepupae that have been found on different substrates.

3. RESULTS: In the sentence "A total of 44 miRNAs (23% of the total microRNAs) showed differential expression between at least two life stage with an LFC" write "stages"

   Corrected Line 128: "A total of 44 miRNAs (23% of the total microRNAs) showed differential expression between at least two stages with an LFC of ±2."

4. DISCUSSION:
   What does it mean "Focusing on arthropod species only"? Which species? I don't understand as the authors said that they performed comparisons with arthropod species.

   We changed the sentence at lines 172-173 as follows: "Focusing our miRNA discovery pipeline on known arthropod species miRNA only, allowed for a stronger comparative analysis of potential microRNA function in BSF."

   "Only three conserved microRNAs were found to be specific to a single life stage: hil-miR-6-2-5p (Egg), hil-miR-3884-3p (Larva), and hil-miR-12418-3p (Pupa)." In the adult stages there are exclusively common microRNAs? Moreover, as the authors identify stage-specific microRNAs, I suggest to perform also a comparison among common microRNAs in all the analysed stages and not exclusively Egg to Larva, Larva to Pupa, Pupa to Female, and Pupa to Male.

   We have changed that sentence to be accurate to the conserved miRNA. An error had occurred.
   Line 189-190: "Seventy-four conserved microRNAs were found to be specific to a single life stage, while only 27 were found across all life stages (S1 Table)."

   We are not sure about the second statement on performing a comparison among common miRNAs in all the "analysed stages

and not exclusively…" As this is a baseline study showing profile of these novel miRNAs, we did not think a comparison of the miRNAs without a high LFC that were found commonly across all life stages would be significant for publication. We included them and their expression values in the supplemental tables, S3 & S4.

"The unique microRNAs identified had a higher number of life stage specific microRNAs: (Egg) hil-miR-m, hil-miR-p, hil-miR-r, hil-miR-s, hil-miR-u, (Pupa) hil-miR-ac, (Female) hil-miR-h, (Male & Female) hil-miR-a, hil-miR-b, and hil-miR-y." This sentence is not really clear: did the adult stage have or not specific microRNAs?

We removed "higher" from the text for clarity at line 191. The adult stages had specific unique miRNAs, but the female adult had one unique miRNA not found in the male adult. We could combine them so it just states "adult" but felt this was more specific.

"However, these 10 unique microRNAs provide insight into the egg, pupa, and adult life stages of the BSF and can be used to compare the developmental regulatory differences of microRNAs to those of other insect species (26)" and what about the larval stage?

Line 196: Added "larva" into the stages.

"In all three cases the females had expression whereas the males had none." What does this sentence mean?

Line 201: We removed this sentence.

"miRNA target prediction" why did the authors choose to explore in detail only 3 microRNAs?

The three explored were selected for their direct implications to mass rearing which we thought had the most impact on the thesis of the paper.
We added the following at line 220: "the target genes discussed below were selected for having the greatest potential impacts on the mass rearing of BSF".

This sentence needs a reference "The microRNA has been seen to increase in expression over the third larval instar, with highest expression levels in the pupa stage, in D. melanogaster."

Line 236: Added reference 44 at the end the sentence.

"Once validated, the microRNA can be harnessed in breeding protocols to improve mass rearing of BSF." please provide the readers concrete ideas of using these microRNA to improve BSF mass rearing.

Lines 249-252: Once validated, the microRNA can be harnessed in breeding protocols to improve mass rearing of BSF by understanding how they adapt to different environments (28, 46, 54) and is being used in the grape and olive industries (55, 56)."

5. METHODS: Please specify the composition of "standardized proprietary diet".

Lines 263-264: The flies were fed *ad libitum* on a standardized proprietary diet based on the commercial composition of layer hen feed.

Why did the authors choose to perform specifically these comparisons? "Five different paired life stages were compared: Egg to Larva, Larva to Pupa, Pupa to Female, and Pupa to Male."

Line 282: We have changed the wording in the methods section by removing the word "paired" to show that we did not only compare Egg to Larva, Larva to Pupa, Pupa to Female, and Pupa to Male. In fact, we did all versus all comparison. The Log fold change of all these comparisons for the Unique miRNA (added as supplementary Table 3 and file labelled S3 Table), and the evolutionary conserved miRNA (added as supplementary Table 4 and file labelled S4 Table).

We have subsequently edited Figure 2 so that it only shows the statistically significant fold changes for the unique miRNAs.

6. FIGURE: "Finally, 21 microRNA were identified with an LFC of ±10 (11% of total miRNA). These included 18 miRNAs conserved across arthropods and 3 miRNAs unique to BSF (Figure 1; Table 3)." In figure 1 only 18 microRNAs were

represented. Please fix this, moreover, looking at the image, I supposed a comparison among all the samples. As I wrote before, it is not clear in the main text.

> **Line 135: We have fixed the figure legend: Figure 1. Heatmap of conserved microRNA expression with a log fold change (LFC) of ±10 (p-adjusted value <0.05) across life stages.**

Thank you,

Alan

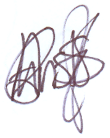

Professor Alan Christoffels PhD, M.ASSAf
Director & DST/NRF Research Chair in Bioinformatics and Health Genomics
MRC Bioinformatics Unit
South African National Bioinformatics Institute
University of the Western Cape
