# Supplementary material for: microRNA profile of Hermetia illucens (black soldier fly) and its implications on mass rearing
Source: PLoS One. 2022 Mar 17;17(3):e0265492. doi: 10.1371/journal.pone.0265492 (PMC8929568; doi:10.1371/journal.pone.0265492)
Supplement: Attachment — Submitted filename: PONE-D-21-33119.docx [file pone.0265492.s005.docx]

**microRNA profile of *Hermetia illucens* (Black soldier fly) and its implications on mass rearing**

**Sarah DeRaedt^1^, Anandi Bierman^2^, Peter van Heusden^1^, Cameron Richards^2^, Alan Christoffels^1^**

**^1^South African National Bioinformatics Institute, South African Medical Research Council Bioinformatics Unit, The University of the Western Cape, Bellville, Western Cape, South Africa**

**^2^AgriProtein Technologies (Pty) Limited, Philippi, Western Cape, South Africa**

**Corresponding Author: Alan Christoffels (**[**alan@sanbi.ac.za**](mailto:alan@sanbi.ac.za)**)**

# ***Abstract***

The growing demands on protein producers and the dwindling available resources have made *Hermetia illucens* (the black soldier fly, BSF) an economically important species. Insights into the genome of this insect will better allow for robust breeding protocols, and more efficient production to be used as a replacement of animal feed protein. The use of microRNA as a method to understand how gene regulation allows insect species to adapt to changes in their environment, has been established in multiple species. The baseline and life stage expression levels established in this study, allow for insight into the development and sex-linked microRNA regulation in BSF. To accomplish this, microRNA was extracted and sequenced from 15 different libraries with each life stage in triplicate. Of the total 192 microRNAs found, 168 were orthologous to known arthropod microRNAs and 24 microRNAs were unique to BSF. Twenty-six of the 168 microRNAs conserved across arthropods had a statistically significant (p < 0.05) differential expression between Egg to Larval stages. The development from larva to pupa was characterized by 16 statistically significant differentially expressed microRNA. Seven and 9 microRNA were detected as statistically significant between pupa to adult female and pupa to adult male, respectively. All life stages had a nearly equal split between up and down regulated microRNAs. Ten of the unique 24 miRNA were detected exclusively in one life stage. The egg life stage expressed five microRNA (*hil-miR-m*, *hil-miR-p, hil-miR-r, hil-miR-s,* and *hil-miR-u)* not seen in any other life stages*.* The female adult and pupa life stages expressed one miRNA each *hil-miR-h* and *hil-miR-ac* respectively. Both male and female adult life stages expressed *hil-miR-a, hil-miR-b,* and *hil-miR-y.* There were no unique microRNAs found only in the larva stage. Twenty-two microRNAs with 56 experimentally validated target genes in the closely related *Drosophila melanogaster* were identified. Thus, the microRNA found display the unique evolution of BSF, along with the life stages and potential genes to target for robust mass rearing. Understanding of the microRNA expression in BSF will further their use in the crucial search for alternative and sustainable protein sources.

**Keywords:** *Hermetia* illucens, Black Soldier Fly, sustainable protein, microRNA expression, life stage, sequencing, mass rearing

# ***Introduction***

The world population is projected to reach 9.8 billion by the year 2050 (1), meaning food production needs to increase by 50% (2). However, food production is expected to decrease by as much as 25% by 2050 due to climate change and unsustainable traditional methods of manufacturing food (2). To combat this decline, new methods of attaining the three major resources involved in food production including food, water, and energy, must be found (3). Due to the increasing demands for protein, it is important to explore sustainable, alternative, large-scale protein sources which are environmentally friendly.

Insects are one alternative source of protein, with an estimated 2,000 species already being consumed mainly in low- and middle-income countries (4). The black soldier fly (*Hermetia illucens;* BSF) is a species found worldwide and can be used as a sustainable animal and fish feed (5, 6). Black soldier fly larvae (BSFL) contain 42% protein, 35% crude fat (7), and 8.72% chitin in prepupae (8). This makes them not only a good protein source, but shows potential for a variety of derived products, and has minimal environmental impacts. Due to these facts, and that they are not a nuisance species, or mechanical disease vector (9, 10), they are an ideal candidate for large scale rearing for food sustainability and waste reduction.

The potential uses for BSF are numerous; however, until recently little has been done to fully understand their genetics and the implications of mass rearing. Many studies have explored the midgut microbiota (11-14). In 2015, a draft genome was published (15), and in 2020 the full genome was sequenced at a depth of 300x coverage (16). Another study still in preprint, has covered the genome using long read PacBio sequencing (17). The midgut transcriptome, and the application of the gene modification tool CRISPR/Cas-9 have also been recently reported for adapting BSF to industrialization (16). Multiple insect genomics studies barring BSF have demonstrated the role of microRNA in regulating biological and behavioural functions at different life stages (18-21). As arthropod genetic diversity is known to diminish through colonization (10, 22), looking directly at gene regulation via microRNA expression explores how the BSF adapts to the mass rearing environment. Mapping the baseline microRNA expression data will provide crucial information on the large-scale development of BSF into an economically sustainable protein source. This study creates a database of novel microRNAs and their expression levels across five life stages and two sexes in BSF and provides candidate microRNAs with potential impact on BSF mass rearing.

# ***RESULTS***

***Sequencing and Mapping***

The microRNA from five different life stages, (egg, larva, pupa, adult unmated female, and adult unmated male) of *H. illucens* were extracted and sequenced to complete a baseline assessment of the species. Illumina TruSeq Small Library platform was used to create the microRNA libraries. The total raw read count for the life stages ranged from 10.6-16.4 million reads with an average sequence length of 51 nucleotides (Table 1). More than 98% of the reads had Phred quality values (PQV) of 20 and 96% with PQV of 30. After the reads were filtered for lengths smaller than 18 nucleotides and any non-canonical values, the average reads were 10.9, 9.1, 11.4, 12.1, and 10.5 million for egg, larva, pupa, adult unmated female, and adult unmated male, respectively.

The percentages of total filtered reads that mapped to the BSF genome were: 3.0% of egg, 0.5% larva, 1.4% pupa, 8.2% female, and 6.4% male libraries. The second larval replicate only had 5.4 million reads after filtering which lowered the overall larval library mapping (Table 1).

**Table 1. Summary of BSF reads sequenced and mapped**.

| **Samples** | **Total Raw Reads** | **Total Filtered Reads** | **Mapped Reads** | **Unmapped Reads** | **Mapped (%)** | **Unmapped (%)** |
| --- | --- | --- | --- | --- | --- | --- |
| 1E | 10634038 | 10485727 | 202996 | 10282731 | 1.9% | 98.1% |
| 2E | 11391345 | 11058752 | 312488 | 10746264 | 2.8% | 97.2% |
| 3E | 11630268 | 11143061 | 471985 | 10671076 | 4.2% | 95.8% |
| E avg | 11218550 | 10895847 | 329156 | 10566690 | 3.0% | 97.0% |
|  |  |  |  |  |  |  |
| 1L | 13716268 | 11013749 | 46243 | 10967506 | 0.4% | 99.6% |
| **2L** | **16363975** | **5374681** | **18318** | **5356363** | **0.3%** | **99.7%** |
| 3L | 14753374 | 1077735 | 74337 | 10703020 | 0.7% | 99.3% |
| L avg | 14944539 | 5822055 | 46299 | 9008963 | 0.5% | 99.5% |
|  |  |  |  |  |  |  |
| 1P | 11717837 | 10156757 | 157402 | 9999355 | 1.5% | 98.5% |
| 2P | 14142734 | 11927696 | 265560 | 11662136 | 2.2% | 97.8% |
| 3P | 12674729 | 11982861 | 89089 | 11893772 | 0.7% | 99.3% |
| P avg | 12845100 | 11355771 | 170684 | 11185088 | 1.5% | 98.5% |
|  |  |  |  |  |  |  |
| 1M | 10958506 | 9495353 | 955239 | 8540114 | 10.1% | 89.9% |
| 2M | 12428650 | 11973265 | 474068 | 11499197 | 4.0% | 96.0% |
| 3M | 10665444 | 10037438 | 527529 | 9509909 | 5.3% | 94.7% |
| M avg | 11350867 | 10502019 | 652279 | 9849740 | 6.5% | 93.5% |
|  |  |  |  |  |  |  |
| 1F | 13126901 | 12795661 | 585428 | 12210233 | 4.6% | 95.4% |
| 2F | 14284210 | 13860109 | 579082 | 13281027 | 4.2% | 95.8% |
| 3F | 11691808 | 9695009 | 1560275 | 8134734 | 16.1% | 83.9% |
| F avg | 13034306 | 12116926 | 908262 | 11208665 | 8.3% | 91.7% |

**Abbreviations: E =egg; L= larva; P= pupae; F=female; M=male; 1-3=replicate; avg=average of 3 replicates**

***Identification of novel microRNAs in Hermetia illucens***

Analysis of the 15 BSF libraries was completed using MiRDeep2 software (v.2.0.1.2), and alignment of all known arthropod microRNAs from miRbase v22.1 was used to discover baseline microRNAs of BSF. As BSF is a novel species with no known/reported microRNA, all microRNA found were considered novel. Any microRNA identified to be conserved across other known arthropods are referred to as conserved, and the microRNA that have yet to be identified in any other species are defined as unique microRNA. In order to be considered unique, both the mature and star sequence needed to be present and found in at least 2 of the 15 libraries. A total of 192 novel microRNAs were found across the 15 BSF libraries. Of these 168 were found to be orthologous to known arthropod microRNAs (S1 Table), and 24 were found to be unique microRNA of BSF (S2 Table), not identified in any other species.

***Differential Expression***

microRNA differential expression was calculated with log fold changes (LFC) of 0, 2, 5, and 10 in expression between life stages of egg to larva, larva to pupa, [upa to adult female, pupa to adult male. Twenty-six of the 168 microRNAs conserved across arthropods had a statistically significant (p-adjusted value < 0.05) differential expression between Egg to Larval stages. Nearly half (14) of these 26 microRNA were downregulated and 12 were upregulated. Development from Larva to Pupa was characterized by 16 statistically significant (p-adjusted value < 0.05) differentially expressed microRNA with equal amount up and downregulated transcripts. Differential expression of seven miRNA were detected as statistically significant (p-adjusted value < 0.05) between Pupa to Adult Female (3 miRNA up regulated and 4 microRNAs down regulated). microRNAs from Pupa to Adult Male had 9 statistically significant expression changes (4 miRNA up regulated and 5 down regulated (Table 2)).

**Table 2. microRNA with statistically significant regulation between life stages of novel BSF microRNA with the same seed as known arthropod microRNA.**

| Life Stage | Egg to Larva | Larva to Pupa | Pupa to Female | Pupa to Male |
| --- | --- | --- | --- | --- |
| Up Regulated | 12 | 8 | 3 | 4 |
| Down Regulated | 14 | 8 | 4 | 5 |
| Total miRNA w/padj<0.05 | 26 | 16 | 7 | 9 |
| Total miRNA | 168 | 168 | 168 | 168 |

A total of 44 miRNAs (23% of the total microRNAs) showed differential expression between at least two life stage with an LFC of ±2. These included 31 miRNAs conserved across all arthropods and 13 unique to BSF. A total of 40 miRNAs (21% of the total microRNAs) showed differential expression between at least two life stages with an LFC of ±5 (30 conserved across all arthropods and 10 unique to BSF). Finally, 21 microRNA were identified with an LFC of ±10 (11% of total miRNA). These included 18 miRNAs conserved across arthropods and 3 miRNAs unique to BSF (Figure 1; Table 3).

**Figure 1. Heatmap of microRNA expression with a log fold change (LFC) of ±10 (p-adjusted value <0.05) across life stages.**

**Table 3. Summary of microRNAs differential expression log fold change (LFC).**

| **Log Fold Change** | **Total miRNA** | **LFC ± 2** | **LFC ± 5** | **LFC ± 10** |
| --- | --- | --- | --- | --- |
| **Known arthropod miRNA** | 168 | 31 | 30 | 18 |
| ***H. illucens* unique** | 24 | 13 | 10 | 3 |
| **Total miRNA** | 192 | 44 | 40 | 21 |

Seven of the unique 24 miRNA were detected exclusively in one life stage (Figure 2). The egg life stage expressed five miRNAs not seen in any other life stages (*hil-miR-m*, *hil-miR-p, hil-miR-r, hil-miR-s,* and *hil-miR-u*; S2 Table)*.* The female adult and pupa life stages expressed one miRNA each (*hil-miR-h* and *hil-miR-ac* respectively; S2 Table). Both male and female adult life stages expressed 3 shared miRNA (*hil-miR-a, hil-miR-b,* and *hil-miR-y;* S2 Table)*.* There were no unique microRNAs found only in the larva stage (Figure 2).

**Figure 2. Heatmap of unique microRNA expression across life stages. Expression is based on average read count of triplicate samples for each life stage.**

***Predicted Target Genes***

BSF miRNAs were searched against experimentally validated target genes in *D. melanogaster* using Flybase.org (FB2020_06) (23) [and MirTarBase (](https://d.docs.live.net/6df12d3134c70d4e/Documents/mirna_study/and MirTarBase ()<http://mirtarbase.cuhk.edu.cn/php/index.php>) (24) to extrapolate BSF genes targeted for miRNA regulation. *D. melanogaster* was chosen as the closest relative to BSF with experimentally validated target genes. These databases represent curated published, and experimentally validated microRNA-Target Gene pathways. Fifty-six genes were found to be targets of 22 BSF microRNAs. The target genes covered a range of developmental functions in Drosophila (Table 4). In the absence of experimentally verified BSF miRNA targets, the closely related insect species (*D. melanogaster)*, provided greater understanding of the development of the fly during rearing. The predicted target genes are characterized by 21 known gene families, the majority identified as C2H2 Zinc Finger Transcription Factors gene family. The second highest representative family were the Basic Helix-Loop-Helix Transcription Factors (negative regulators of notch signaling pathway), followed by RHG Proteins family. The Bearded Gene Family and ABCG ATP-Binding Cassette Transporter Subfamily matched two genes each. The other 16 gene families were each represented by single genes. Each of these genes were targeted by multiple microRNAs.

**Table 4. Novel H. illucens microRNA with orthologous microRNA and their predicted target genes in *Drosophila*.**

| **Novel *H. illucens* miRNA with same seed** | **Known Predicted Target Gene in *Drosophila*** | **Egg to Larva**  **LFC*** | **Larva to Pupa**  **LFC*** | **Pupa to Female**  **LFC*** | **Pupa to Male**  **LFC*** |
| --- | --- | --- | --- | --- | --- |
| hil-bantam-3p | W | 2.563 | -0.926 | -0.78 | -1.172 |
| hil-let-7-5p | ab | 2.074 | 8.84 | 0.832 | 0.192 |
| hil-miR-124-3p | ana, gli | -4.198 | 5.269 | 1.485 | 0.65 |
| hil-miR-1-3p | Amyotrophic lateral sclerosis 2, CG11377, CG17065, CG18542, CG31121, Chd64, crim, DI, Jafrac2, Msr-110, Nedd4, sinu, tutl | 0.403 | 0.48 | -1.166 | -1.493 |
| hil-miR-14-3p | EcR, Ice, IP3K2, sug | 1.84 | 0.078 | -0.675 | -0.828 |
| hil-miR-263a-5p | W | -0.335 | 2.089 | 1.466 | 1.184 |
| hil-miR-276a-3p | DopR | 1.816 | 2.127 | 0.417 | 0.008 |
| hil-miR-278-3p | ex, tup | 10.304 | -0.466 | 2.175 | 1.362 |
| hil-miR-279-3p | esg, nerfin-1, os, SP555 | -3.171 | 0.839 | 0.388 | 0.604 |
| hil-miR-283-5p | cos, smo | 1.562 | -2.857 | -0.412 | -0.267 |
| hil-miR-2a-3p | grim, reaper, skl, hid, malpha, HLHmdelta | -3.466 | 1.563 | 0.821 | 0.693 |
| hil-miR-315-5p | Axn, Notum | -1.824 | 1.066 | 0.843 | 0.772 |
| hil-miR-316-5p | IA-2 | 7.816 | 0.174 | 2.418 | 1.813 |
| hil-miR-317-3p | yellow-c | 1.164 | -1.118 | 1.486 | 14.041 |
| hil-miR-34-5p | Eip74EF, Su(z)12 | 6.163 | -6.769 | -6.126 | -5.636 |
| hil-miR-5-5p | smo | 2.074 | 0 | -4.82 | -6.715 |
| hil-miR-7-5p | e, fng, h, HLHm5, I(1)MZ4, iHog, ttk | -9.097 | 3.818 | -0.892 | 0.181 |
| hil-miR-8-3p | CG13060, CG32767, CG8420, Cpr56F, ena, Gug, pan, ush, wls | 2.353 | -0.304 | -0.043 | 0.173 |
| hil-miR-92a-3p | sha | -7.694 | 2.342 | 0.993 | 2.052 |
| hil-miR-9a-5p | Bx, dg, sens | -2.725 | -0.306 | 1.621 | 1.746 |
| hil-miR-iab-4-5p | abd-A, Ubx | -6.179 | 4.019 | -0.978 | 1.564 |
| hil-miR-iab-8-5p | abd-A, Abd-B, Ubx | 2.074 | 0 | 0 | -7.464 |

*LFC = Log Fold Change between life stages listed

# ***DISCUSSION***

BSF has become an insect of great economic and environmental value over the past decades. This study established a baseline survey of novel microRNAs, both conserved and unique, in the BSF and their expression levels across 5 different life stages and identified likely target genes for these miRNAs. Like most species, the BSF genome retains a highly conserved microRNA library as evidenced by the large numbers of orthologous microRNAs found from a wide range of arthropod species in the miRBase database. Focusing on arthropod species only, allowed for closer comparative analysis of potential microRNA function in BSF. Finding orthologs of microRNAs across species results in a higher power of discovery for *de novo* microRNAs (25). The high number of orthologs found for each microRNA family, along with the strict identification criterion utilized by the miRDeep2 program for mature, star, and precursor sequences, provide high confidence in the microRNAs found in the BSF genome. These new BSF microRNA add another species that follow the highly conserved nature of microRNA across all species.

Overall, 168 novel microRNAs were found in the black soldier fly, with the vast majority conserved (87%). The 24 unique microRNAs that passed the criteria to be included, came from a group of 91 potentially unique sequences found. The criteria used ensured that reporting would only include highly likely candidates. However, the many sequences not considered candidates, represent a potential larger network of microRNAs for future study. The limited number of libraries per life stage (3) could mean some candidate sequences were missed. BSF has already been seen to have a divergent and large genomic landscape (16), and the number of microRNAs follows suit.

***Stage specific miRNA expression***

Understanding the basic expression levels across the life stages has led to a better understanding of stage specific microRNA regulation. Only three conserved microRNAs were found to be specific to a single life stage: *hil-miR-6-2-5p* (Egg), *hil-miR-3884-3p* (Larva), and *hil-miR-12418-3p* (Pupa). These three may be important for gene regulation linked to life stage specific development. The unique microRNAs identified had a higher number of life stage specific microRNAs: (Egg) *hil-miR-m*, *hil-miR-p, hil-miR-r, hil-miR-s, hil-miR-u,* (Pupa) *hil-miR-ac*, (Female) *hil-miR-h*, (Male & Female) *hil-miR-a, hil-miR-b,* and *hil-miR-y.* These 10 represent nearly half (42%) of the unique microRNAs found. This higher life stage specificity may be due to too low frequencies of the fragments to be detected through sequencing in the other life stages. However, these 10 unique microRNAs provide insight into the egg, pupa, and adult life stages of the BSF and can be used to compare the developmental regulatory differences of microRNAs to those of other insect species (26) . The inclusion of separate male and female life stages allows insight into the sex-linked microRNAs of BSF, and their potential functions in development in sex differentiation (27, 28). For instance, *hil-miR-h, hil-miR-317-3p, hil-miR-3805a-5p,* and *hil-miR-2548-3p* displayed different expression levels between female and male samples. In all three cases the females had expression whereas the males had none. The unique microRNA of *hil-miR-af* was found in the larval and male stages, but not the female life stage. These stage specific microRNAs provide valuable developmental information unique to BSF showing their genetic adaptability and divergence from other insect species (16, 29, 30).

Eighteen conserved microRNAs were found with substantial (LFC 10) expression regulation between at least two life stages. While these do not have any known experimentally validated predicted target genes, the level of differential expression makes them microRNA of interest for future study.

***miRNA target prediction***

Looking at the functions of possible target genes highlights how important the regulation of microRNA is in the development of insects. While most of the predicted target genes deal with cell development, differentiation, and death; the target genes discussed below have potential impacts on the mass rearing of BSF.

*bantam-3p* has been well established in *D. melanogaster* and involved in the cell differentiation, apoptosis, neural development, and germ line maintenance (31, 32). The microRNA is expressed during the 3^rd^ larval instar stage, regulating optical disc and photo receptor differentiation (32). b*antam* is linked to mediating the circadian rhythm proteins (*clk*) in *D. melanogaster*. Overexpression of *bantam* causes a lengthening of the circadian period (33). BSF *bantam* expression follows a path consistent with the developmental role it has been identified to play with higher expression in larva, followed by a decreasing trend from larva to pupa, and both adult stages where it was heavily downregulated. Both factors may be exploited for optimal mass rearing as BSF have been shown to have a direct relationship between length of light exposure and egg production (34).

The *let-7* microRNA is one of the first to be identified in *C. elegans* and is known to be highly conserved across most species (35). Controlling expression of *let-7* is essential to prevent deleterious phenotypes such as wing, fertility, motility, and flight deficiencies from unusual abdominal musculature maturation (35-39). *let-*7 has been shown to regulate the ecdysis pathways during molting stages in ticks (40) and silkworms (41). The microRNA has been seen to increase in expression over the third larval instar, with highest expression levels in the pupa stage, in *D. melanogaster*. BSF *let-*7 follows the same expression pattern, peaking in expression during the pupal stage.

Overexpression of *mir-14* leads to lean *D. melanogaster* (42). *Sugarbabe* is one of the target genes for *mir-14* and has been shown to be controlled by both diet nutrient levels and *mir-14* targeting in the face of nutrient deprivation (43). The non-nutrient dependent nature of *mir-14* regulation has led to flies being obese during starvation (43). As BSFL fat content is a known beneficial part of the role of BSF as sustainable feed additive (44), being able to potentially increase fat content would be a useful tool in the mass rearing process.

***Future work***

The baseline of conserved and unique novel microRNAs in the BSF genome were all identified by utilizing established computational methods. As BSF is a novel species with a relatively new genome, the baseline data identified here should be confirmed through laboratory validation. These microRNA sequences should be used to confirm the predicted target genes and regulatory pathways. Once validated, the microRNA can be harnessed in breeding protocols to improve mass rearing of BSF.

***Conclusions***

The conserved and unique microRNA described in this study form an essential understanding of gene regulation in the economically and environmentally important species of *H. illucens* and will provide potential targets for genetic manipulation of this species in order to improve its use as an alternative protein source.

***Acknowledgements***

Eugene DeBeste is acknowledged for his support with software and server acquisition and maintenance.

# ***Methods***

***Sample Collection***

*Hermetia illucens* (black soldier fly, BSF) were reared under factory breeding conditions by AgriProtein Technologies Ltd in Philippi, South Africa. The flies were fed *ad libitum* on a standardized proprietary diet. All life stages were kept at 28°C (±2°C), 80% relative humidity, under 12-hour day and night cycles.

Fifteen specimens were selected across five life stages: egg, larva, pupa, adult unmated female, and adult unmated male. The 15 specimens were triplicates for each of the five life stages. In order to acquire enough material to extract microRNAs according to methodology, an egg batch laid from a single female was used for each egg replicate. The egg batches were laid one day prior to collection. Larvae were collected during the L5 instar, which was defined as day 21 (±2) from egg. The L5 instar was selected due to its size and ease of identification, along with the life stage’s importance in protein production. The pupal stage was defined as day 28 (±2) from egg. Adult females and males were collected before being allowed to mate.

***microRNA Extraction***

All specimens were harvested on the same day and transported to the lab in sterile falcon tubes. The flies were left to acclimate to the lab for 60 minutes after transport to allow any stress from travel to reduce. The samples were then flash frozen with liquid nitrogen and ground into a powder. The lysing agent QIAzol was used, and the microRNA extraction kit miRNeasy Mini Kit from Qiagen (45) standard protocols were followed. A Qbit RNA Assay (Invitrogen™) was performed for quality control and to ensure enough microRNA was extracted for sequencing. After extraction, all samples were stored at -80°C. To ship samples for sequencing, the microRNA was stabilized at room temperature using RNAstable^®^ (Biomatrica^®)^.

***Sequencing***

Extracted samples were shipped to Macrogen Inc (Seoul, South Korea) for small RNA sequencing. Illumina TruSeq Small RNA Library construction of the 15 samples was completed. Sequencing was done on an Illumina HiSeq 2500 machine with 8 million reads per sample.

***Read Processing and Analysis***

Sequences from the 15 different libraries were quality checked using FastQC (http://www bioinformatics.babraham.ac.uk/projects/fastqc/)(46). Reads were filtered for any non-canonical letters, and the 3’ adapters were trimmed, followed by the removal of any reads shorter than 18 nucleotides long using the MiRDeep2 (v.2.0.1.2) program (47). The genome of *Hermetia illucens* was used to map the reads (NCBI: assembly iHerIll2.2.curated20191125). The genome was indexed for mapping using Bowtie (v1.1.1)(48).

After mapping the reads, MiRDeep2 was used to identify any exact matches to published microRNAs (miRBase, v22.1) (49-56) found in the sample reads. Initially, all known microRNAs from miRBase were used, then filtered for arthropod species only for more closely related relevance of the microRNAs. The microRNAs found were given read counts using the Quantifier.pl script from MiRDeep2.

***Differential Expression***

Differential expression was calculated using the DESeq2 (57) package in the R programming language. Expression was calculated based on read counts that were normalized using DeSeq2 statistical program. DeSeq2 normalizes read counts for sequencing depth and RNA composition. Five different paired life stages were compared: Egg to Larva, Larva to Pupa, Pupa to Female, and Pupa to Male. DESeq2 utilized a negative binomial generalized linear model to test for statistical significance in expression between the life stages. Raw read counts are normalized using a median of ratios method which accounts for sequencing depth and RNA composition. Therefore, read counts were normalized for more than reads per million to account for differences in sequencing quality and length of microRNAs. All analysis was completed using R (58-60).

References

1.	United Nations Department of Economic and Social Affairs PD. World Population Prospects The 2017 Revision: Key Findings and Advance Tables. United Nations; 2017. Report No.: ESA/P/WP/248.

2.	Nations FaAOotU. The State of Food and Agriculture: Climate Change, Agriculture and Food Security. Rome: United Nations; 2016. Report No.: 0081-4539.

3.	Trines E, Hohne N, Jung M, Skutsch M, Petsonk A, Silva-Chavez G, et al. Climante Change Scientific Assessment and Policy Analysis. Netherlands: Netherlands Environmental Assessment Agency; 2006. Contract No.: 500102002.

4.	Ramos-Elorduy J, Gonzalez EA, Hernandez AR, Pino JM. Use of *Tenebrio molitor* (Coleoptera: Tenebrionidae) to Recycle Organic Wastes and as Feed for Broiler Chickens. Veterinary Entomology. 2002;95(1):214-20.

5.	Hale OM. Dried Hermetia illucens (italic) larvae (Diptera: Stratiomyidae) as a feed additive for poultry. Journal of the Georgia Entomological Society 2013;8:16-20.

6.	Bondari K, Sheppard DC. Soldier Fly Larvae as Feed in Commercial Fish Production. Aquaculture. 1981;24:103-9.

7.	Jr. BCV, G.L. N, O.M. H, R.W B. Manure residue as a substrate for protein production via Hermetia illucens (italic) larvae [for swine rations]. Proceedings of the Cornell Agricultural Waste Management Conference. 1977:599-604.

8.	Diener S, Zurbrugg C, Tockner K. Conversion of organic material by black soldier fly larvae: establishing optimal feeding rates. Waste Manag Res. 2009;27(6):603-10.

9.	Furman DP, Young RD, Catts EP. *Hermetia illucens* (Linnaeus) as a Factor in the Natural Control of *Musca domestica* Linnaeus. Journal of Economic Entomology. 1959;52(5):917-21.

10.	Lainhart W, Conn JE, Rios CT, Vinetz JM, Bickersmith SA, Moreno M. Changes in Genetic Diversity from Field to Laboratory During Colonization of Anopheles darlingi (italic) Root (Diptera: Culicidae). The American Journal of Tropical Medicine and Hygiene. 2015;93(5):998-1001.

11.	De Smet J, Wynants E, Cos P, Campenhout LV. Microbial Community Dynamics during Rearing of Black Soldier Fly Larvae (*Hermetia illucens)* and Impact on Exploitation Potential. Applied and Environmental Microbiology. 2018;84(9).

12.	Jiang CL, Jin WZ, Tao XH, Zhang Q, Zhu J, Feng SY, et al. Black soldier fly larvae (Hermetia illucens) (italic) strengthen the metabolic function of food waste biodegradation by gut microbiome. Microb Biotechnol. 2019;12(3):528-43.

13.	Bonelli M, Bruno D, Brilli M, Gianfranceschi N, Tian L, Tettamanti G, et al. Black Soldier Fly Larvae Adapt to Different Food Substrates through Morphological and Functional Responses of the Midgut. Int J Mol Sci. 2020;21(14).

14.	Bruno D, Bonelli M, Cadamuro AG, Reguzzoni M, Grimaldi A, Casartelli M, et al. The digestive system of the adult Hermetia illucens (italic) (Diptera: Stratiomyidae): morphological features and functional properties. Cell Tissue Res. 2019;378(2):221-38.

15.	Vicoso B, Bachtrog D. Numerous transitions of sex chromosomes in Diptera. PLoS Biol. 2015;13(4):e1002078.

16.	Zhan S, Fang G, Cai M, Kou Z, Xu J, Cao Y, et al. Genomic landscape and genetic manipulation of the black soldier fly Hermetia illucens (italic), a natural waste recycler. Cell Res. 2020;30(1):50-60.

17.	Generalovic TN, McCarthy SA, Warren IA, Wood JMD, Torrance J, Sims Y, et al. A high-quality, chromosome-level genome assembly of the Black Soldier Fly (*Hermetia Illucens* L.). bioRxiv preprint. 2020.

18.	Behura SK. Insect microRNAs: Structure, function and evolution. Insect Biochem Mol Biol. 2007;37(1):3-9.

19.	Asgari S. MicroRNA functions in insects. Insect Biochem Mol Biol. 2013;43(4):388-97.

20.	Lucas KJ, Myles KM, Raikhel AS. Small RNAs: a new frontier in mosquito biology. Trends Parasitol. 2013;29(6):295-303.

21.	Wei Y, Chen S, Yang P, Ma Z, Kang L. Characterization and comparative profiling of the small RNA transcriptomes in two phases of locust. Genome Biol. 2009;10(1):R6.

22.	Godinho DP, Cruz MA, Charlery De La Masselière M, Teodoro‐Paulo J, Eira C, Fragata I, et al. Creating outbred and inbred populations in haplodiploids to measure adaptive responses in the laboratory. Ecology and Evolution. 2020;10(14):7291-305.

23.	Larkin A, Marygold SJ, Antonazzo G, Attrill H, Dos Santos G, Garapati PV, et al. FlyBase: updates to the Drosophila melanogaster (italic) knowledge base. Nucleic Acids Res. 2021;49(D1):D899-D907.

24.	Huang HY, Lin YC, Li J, Huang KY, Shrestha S, Hong HC, et al. miRTarBase 2020: updates to the experimentally validated microRNA-target interaction database. Nucleic Acids Res. 2020;48(D1):D148-D54.

25.	Stark A, Kheradpour P, Parts L, Brennecke J, Hodges E, Hannon GJ, et al. Systematic discovery and characterization of fly microRNAs using 12 Drosophila genomes. Genome Res. 2007;17(12):1865-79.

26.	Ylla G, Piulachs M-D, Belles X. Comparative analysis of miRNA expression during the development of insects of different metamorphosis modes and germ-band types. BMC Genomics. 2017;18(1).

27.	Peng W, Tariq K, Xie J, Zhang H. Identification and Characterization of Sex-Biased MicroRNAs in Bactrocera dorsalis (italic) (Hendel). PLoS One. 2016;11(7):e0159591.

28.	Zhang X, Zheng Y, Jagadeeswaran G, Ren R, Sunkar R, Jiang H. Identification and developmental profiling of conserved and novel microRNAs in Manduca sexta (italic). Insect Biochemistry and Molecular Biology. 2012;42(6):381-95.

29.	Ylla G, Fromm B, Piulachs M-D, Belles X. The microRNA toolkit of insects. Scientific Reports. 2016;6(1):37736.

30.	Lucas KJ, Zhao B, Liu S, Raikhel AS. Regulation of physiological processes by microRNAs in insects. Current Opinion in Insect Science. 2015;11:1-7.

31.	Brennecke J, Hipfner DR, Stark A, Russell RB, Cohen SM. *bantam* Encodes a Developmentally Regulated microRNA that Controls Cell Proliferatino and regulates the Proapoptotic Gene *hid* in *Drosophila*. Cell. 2003;113(1):25-36.

32.	Li Y, Padgett RW. bantam is required for optic lobe development and glial cell proliferation. PLoS One. 2012;7(3):e32910.

33.	Kadener S, Menet JS, Sugino K, Horwich MD, Weissbein U, Nawathean P, et al. A role for microRNAs in the Drosophila circadian clock. Genes Dev. 2009;23(18):2179-91.

34.	Hoc B, Noel G, Carpentier J, Francis F, Caparros Megido R. Optimization of black soldier fly (Hermetia illucens) (italic) artificial reproduction. PLoS One. 2019;14(4):e0216160.

35.	Pasquinelli A, Reinhart B, Slack F, Martindale M, Kuroda M, Maller B, et al. Conservation of the sequence and temporal expression of *let-7* heterochronic regulatory RNA. Nature. 2000;408:86-9.

36.	Lee H, Han S, Kwon CS, Lee D. Biogenesis and regulation of the let-7 miRNAs and their functional implications. Protein Cell. 2016;7(2):100-13.

37.	Caygill EE, Johnston LA. Temporal regulation of metamorphic processes in Drosophila by the let-7 and miR-125 heterochronic microRNAs. Curr Biol. 2008;18(13):943-50.

38.	Sokol NS, Xu P, Jan YN, Ambros V. Drosophila let-7 microRNA is required for remodeling of the neuromusculature during metamorphosis. Genes Dev. 2008;22(12):1591-6.

39.	Hertel J, Bartschat S, Wintsche A, Otto C, Students of the Bioinformatics Computer L, Stadler PF. Evolution of the let-7 microRNA family. RNA Biol. 2012;9(3):231-41.

40.	Wu F, Luo J, Chen Z, Ren Q, Xiao R, Liu W, et al. MicroRNA let-7 regulates the expression of ecdysteroid receptor (ECR) in Hyalomma asiaticum (italic) (Acari: Ixodidae) ticks. Parasit Vectors. 2019;12(1):235.

41.	Ling L, Ge X, Li Z, Zeng B, Xu J, Aslam AF, et al. MicroRNA Let-7 regulates molting and metamorphosis in the silkworm, Bombyx mori (italic). Insect Biochem Mol Biol. 2014;53:13-21.

42.	Xu P, Vernooy SY, Guo M, Hay BA. The Drosophila MicroRNA Mir-14 Suppresses Cell Death and Is Required for Normal Fat Metabolism. Current Biology. 2003;13(9):790-5.

43.	Varghese J, Lim SF, Cohen SM. Drosophila miR-14 regulates insulin production and metabolism through its target, sugarbabe. Genes Dev. 2010;24(24):2748-53.

44.	Kim YB, Kim DH, Jeong SB, Lee JW, Kim TH, Lee HG, et al. Black soldier fly larvae oil as an alternative fat source in broiler nutrition. Poult Sci. 2020;99(6):3133-43.

45.	Qiagen. <miRNeasy-Mini-Handbook-WW.pdf>.

46.	FastQC. 2015.

47.	Friedlander MR, Mackowiak SD, Li N, Chen W, Rajewsky N. miRDeep2 accurately identifies known and hundreds of novel microRNA genes in seven animal clades. Nucleic Acids Res. 2012;40(1):37-52.

48.	Langmead B, Trapnell C, Pop M, Salzberg SL. Ultrafast and memory-efficient alignment of short DNA sequences to the human genome. Genome Biol. 2009;10(3):R25.

49.	Kozomara A, Birgaoanu M, Griffiths-Jones S. miRBase: from microRNA sequences to function. Nucleic Acids Res. 2019;47(D1):D155-D62.

50.	Kozomara A, Griffiths-Jones S. miRBase: annotating high confidence microRNAs using deep sequencing data. Nucleic Acids Res. 2014;42(Database issue):D68-73.

51.	Kozomara A, Griffiths-Jones S. miRBase: integrating microRNA annotation and deep-sequencing data. Nucleic Acids Res. 2011;39(Database issue):D152-7.

52.	Ambros V, Bartel B, Bartel DP, Burge CB, Carrington JC, Chen X, et al. A uniform system for microRNA annotation. RNA. 2003;9(3):277-9.

53.	Griffiths-Jones S. The microRNA Registry. Nucleic Acids Res. 2004;32(Database issue):D109-11.

54.	Griffiths-Jones S, Bateman A, Marshall M, Khanna A, Eddy SR. Rfam: an RNA family database. Nucleic Acids Res. 2003;31(1):439-41.

55.	Griffiths-Jones S, Grocock RJ, van Dongen S, Bateman A, Enright AJ. miRBase: microRNA sequences, targets and gene nomenclature. Nucleic Acids Res. 2006;34(Database issue):D140-4.

56.	Griffiths-Jones S, Saini HK, van Dongen S, Enright AJ. miRBase: tools for microRNA genomics. Nucleic Acids Res. 2008;36(Database issue):D154-8.

57.	Love MI, Huber W, Anders S. Moderated estimation of fold change and dispersion for RNA-seq data with DESeq2. Genome Biol. 2014;15(12):550.

58.	Team RC. R: A language and environment for statistical computing. Vienna, Austria: R Foundation for Statistical Computing; 2017.

59.	Wickham H. ggplot2: Elegant Graphics for Data Analysis. New York: Springer-Verlag; 2009.

60.	Wickham H, Averick M, Bryan J, Chang W, McGowan L, François R, et al. Welcome to the Tidyverse. Journal of Open Source Software. 2019;4(43).
