# Supplementary material for: microRNA profile of Hermetia illucens (black soldier fly) and its implications on mass rearing
Source: PLoS One. 2022 Mar 17;17(3):e0265492. doi: 10.1371/journal.pone.0265492 (PMC8929568; doi:10.1371/journal.pone.0265492)
Supplement: Peer review history

# Peer review: Decision Letter 0

**DOI:** 10.1371/journal.pone.0265492.r001

**Authors:** Patrizia Falabella

**Transfer Alert**

This paper was transferred from another journal. As a result, its full editorial history (including decision letters, peer reviews and author responses) may not be present.

23 Nov 2021

PONE-D-21-33119microRNA profile of Hermetia illucens (Black Soldier Fly) and implications on mass rearingPLOS ONE

Dear Dr. Christoffels,

Thank you for submitting your manuscript to PLOS ONE. After careful consideration, we feel that it has merit but does not fully meet PLOS ONE’s publication criteria as it currently stands. Therefore, we invite you to submit a revised version of the manuscript that addresses the points raised during the review process.

Additional Editor Comments:

Your manuscript entitled has now been reviewed, and the reviewer comments are appended below and in a separate word file. You will see that, while they find your work of interest, they have raised points that need to be addressed before we can make a decision on publication. Please submit your revised manuscript by Jan 7, 2022. If you will need more time than this to complete your revisions, please reply to this message or contact the journal office at plosone@plos.org. When you're ready to submit your revision, log on to https://www.editorialmanager.com/pone/ and select the 'Submissions Needing Revision' folder to locate your manuscript file.

Please include the following items when submitting your revised manuscript:

- A rebuttal letter that responds to each point raised by the academic editor and reviewer(s). You should upload this letter as a separate file labeled 'Response to Reviewers'.
- A marked-up copy of your manuscript that highlights changes made to the original version. You should upload this as a separate file labeled 'Revised Manuscript with Track Changes'.
- An unmarked version of your revised paper without tracked changes. You should upload this as a separate file labeled 'Manuscript'.

If you would like to make changes to your financial disclosure, please include your updated statement in your cover letter. Guidelines for resubmitting your figure files are available below the reviewer comments at the end of this letter.

If applicable, we recommend that you deposit your laboratory protocols in protocols.io to enhance the reproducibility of your results. Protocols.io assigns your protocol its own identifier (DOI) so that it can be cited independently in the future. For instructions see: https://journals.plos.org/plosone/s/submission-guidelines#loc-laboratory-protocols. Additionally, PLOS ONE offers an option for publishing peer-reviewed Lab Protocol articles, which describe protocols hosted on protocols.io. Read more information on sharing protocols at https://plos.org/protocols?utm_medium=editorial-email&utm_source=authorletters&utm_campaign=protocols.

We look forward to receiving your revised manuscript.

Kind regards,

Patrizia Falabella

Academic Editor

PLOS ONE

Journal Requirements:

When submitting your revision, we need you to address these additional requirements.

1. Please ensure that your manuscript meets PLOS ONE's style requirements, including those for file naming. The PLOS ONE style templates can be found at 

https://journals.plos.org/plosone/s/file?id=wjVg/PLOSOne_formatting_sample_main_body.pdf and 

https://journals.plos.org/plosone/s/file?id=ba62/PLOSOne_formatting_sample_title_authors_affiliations.pdf.

2. We note that you have stated that you will provide repository information for your data at acceptance. Should your manuscript be accepted for publication, we will hold it until you provide the relevant accession numbers or DOIs necessary to access your data. If you wish to make changes to your Data Availability statement, please describe these changes in your cover letter and we will update your Data Availability statement to reflect the information you provide.

3. Please include captions for your Supporting Information files at the end of your manuscript, and update any in-text citations to match accordingly. Please see our Supporting Information guidelines for more information: http://journals.plos.org/plosone/s/supporting-information. 

[Note: HTML markup is below. Please do not edit.]

Reviewers' comments:

Reviewer's Responses to Questions

**Comments to the Author**

1. Is the manuscript technically sound, and do the data support the conclusions?

The manuscript must describe a technically sound piece of scientific research with data that supports the conclusions. Experiments must have been conducted rigorously, with appropriate controls, replication, and sample sizes. The conclusions must be drawn appropriately based on the data presented. 

Reviewer #1: Yes

Reviewer #2: Yes

**********

2. Has the statistical analysis been performed appropriately and rigorously? 

Reviewer #1: Yes

Reviewer #2: Yes

**********

3. Have the authors made all data underlying the findings in their manuscript fully available?

The PLOS Data policy requires authors to make all data underlying the findings described in their manuscript fully available without restriction, with rare exception (please refer to the Data Availability Statement in the manuscript PDF file). The data should be provided as part of the manuscript or its supporting information, or deposited to a public repository. For example, in addition to summary statistics, the data points behind means, medians and variance measures should be available. If there are restrictions on publicly sharing data—e.g. participant privacy or use of data from a third party—those must be specified.

Reviewer #1: Yes

Reviewer #2: Yes

**********

4. Is the manuscript presented in an intelligible fashion and written in standard English?

PLOS ONE does not copyedit accepted manuscripts, so the language in submitted articles must be clear, correct, and unambiguous. Any typographical or grammatical errors should be corrected at revision, so please note any specific errors here.

Reviewer #1: No

Reviewer #2: Yes

**********

5. Review Comments to the Author

Please use the space provided to explain your answers to the questions above. You may also include additional comments for the author, including concerns about dual publication, research ethics, or publication ethics. (Please upload your review as an attachment if it exceeds 20,000 characters)

Reviewer #1: Dear Prof. Falabella

The manuscript with ID PONE-D-21-33119 by DeRaedt and coauthors studied the microRNA profile of Hermetia illucens and its implications on mass rearing. The manuscript is interesting, tested hypothesis is well-defined and methodologies are well-described. However, I found numerous revisions that should be carefully revised by authors before the manuscript being considered for publication in PLOS ONE.

Please find the attached word file with some track changes.

Line 57: Support with The feasibility of using yellow mealworms (Tenebrio molitor): Towards a sustainable aquafeed industry. Animals, 2021, 11(3), pp. 1–38, 811

Line 58: Support with

Aquaculture, 2020, 522, 735136

Fish and Shellfish Immunology, 2021, 111, pp. 111–118

All scientific names present in the references should be written italic (Please see the attached word file).

Reviewer #2: In this paper authors analyzed the profile of microRNAs in all the developmental stages of Hermetia illucens. The aim was to relate them to possible functions in order to understand, in future works, the specific gene regulation to improve mass rearing techniques. I have some doubts that must be addressed, especially regarding the comparison among different life stages, it is not clear, and it seems that in many points sentences seem to be contradictory.

KEYWORDS

I suggest to change the keywords that are already in the title

INTRODUCTION

“Black soldier fly larvae (BSFL) contain 42% protein, 35% crude fat (7), and 8.72% chitin in prepupae (8).” This sentence is too generic, protein, lipid and even chitin content change according to the feeding substrates. I suggest to report some examples of different substrates and a range of protein, lipid and chitin content (for example https://doi.org/10.1038/s41598-019-46603-z; https://doi.org/10.1007/s12649-019-00924-2; https://doi.org/10.3390/su131810198)

Authors wrote “they are an ideal candidate for large scale rearing for food sustainability and waste reduction.” Although few studies are performed at industrial scale, I suggest to cite them (for example https://doi.org/10.1038/s41598-020-76571-8; https://doi.org/10.3390/ani10071243)

RESULTS

In the sentence “A total of 44 miRNAs (23% of the total microRNAs) showed differential expression between at least two life stage with an LFC” write “stages”

DISCUSSION

What does it mean “Focusing on arthropod species only”? Which species? I don’t understand as the authors said that they performed comparisons with arthropod species.

“Only three conserved microRNAs were found to be specific to a single life stage: hil-miR-6-2-5p (Egg), hil-miR-3884-3p (Larva), and hil-miR-12418-3p (Pupa).” In the adult stages there are exclusively common microRNAs? Moreover, as the authors identify stage-specific microRNAs, I suggest to perform also a comparison among common microRNAs in all the analysed stages and not exclusively Egg to Larva, Larva to Pupa, Pupa to Female, and Pupa to Male.

“The unique microRNAs identified had a higher number of life stage specific microRNAs: (Egg) hil-miR-m, hil-miR-p, hil-miR-r, hil-miR-s, hil-miR-u, (Pupa) hil-miR-ac, (Female) hil-miR-h, (Male & Female) hil-miR-a, hil-miR-b, and hil-miR-y.” This sentence is not really clear: did the adult stage have or not specific microRNAs?

“However, these 10 unique microRNAs provide insight into the egg, pupa, and adult life stages of the BSF and can be used to compare the developmental regulatory differences of microRNAs to those of other insect species (26)” and what about the larval stage?

“In all three cases the females had expression whereas the males had none.” What does this sentence mean?

“miRNA target prediction” why did the authors choose to explore in detail only 3 microRNAs?

This sentence needs a reference “The microRNA has been seen to increase in expression over the third larval instar, with highest expression levels in the pupa stage, in D. melanogaster.”

“Once validated, the microRNA can be harnessed in breeding protocols to improve mass rearing of BSF.” please provide the readers concrete ideas of using these microRNA to improve BSF mass rearing.

METHODS

Please specify the composition of “standardized proprietary diet”.

Why did the authors choose to perform specifically these comparisons? “Five different paired life stages were compared: Egg to Larva, Larva to Pupa, Pupa to Female, and Pupa to Male.”

FIGURE

“Finally, 21 microRNA were identified with an LFC of ±10 (11% of total miRNA). These included 18

miRNAs conserved across arthropods and 3 miRNAs unique to BSF (Figure 1; Table 3).” In figure 1 only 18 microRNAs were represented. Please fix this, moreover, looking at the image, I supposed a comparison among all the samples. As I wrote before, it is not clear in the main text.

**********

6. PLOS authors have the option to publish the peer review history of their article (what does this mean?). If published, this will include your full peer review and any attached files.

If you choose “no”, your identity will remain anonymous but your review may still be made public.

**Do you want your identity to be public for this peer review?** For information about this choice, including consent withdrawal, please see our Privacy Policy.

Reviewer #1: No

Reviewer #2: No

[NOTE: If reviewer comments were submitted as an attachment file, they will be attached to this email and accessible via the submission site. Please log into your account, locate the manuscript record, and check for the action link "View Attachments". If this link does not appear, there are no attachment files.]

While revising your submission, please upload your figure files to the Preflight Analysis and Conversion Engine (PACE) digital diagnostic tool, https://pacev2.apexcovantage.com/. PACE helps ensure that figures meet PLOS requirements. To use PACE, you must first register as a user. Registration is free. Then, login and navigate to the UPLOAD tab, where you will find detailed instructions on how to use the tool. If you encounter any issues or have any questions when using PACE, please email PLOS at figures@plos.org. Please note that Supporting Information files do not need this step.

Attachment Submitted filename: PONE-D-21-33119.docx Click here for additional data file.

# Author comment: Author response to Decision Letter 0

**DOI:** 10.1371/journal.pone.0265492.r002

28 Feb 2022

Please find below our responses to each of the Reviewers’ comments. Our responses are in bold font.

Reviewer #1: Dear Prof. Falabella

The manuscript with ID PONE-D-21-33119 by DeRaedt and coauthors studied the microRNA profile of Hermetia illucens and its implications on mass rearing. The manuscript is interesting, tested hypothesis is well-defined and methodologies are well-described. However, I found numerous revisions that should be carefully revised by authors before the manuscript being considered for publication in PLOS ONE.

Thank you so much for your review and comments. We have gone through and made the changes and edits you have suggested. 

1. Please find the attached word file with some track changes.

We have gone through and corrected each of the track changes found in the attached document.

2. Line 57: Support with The feasibility of using yellow mealworms (Tenebrio molitor): Towards a sustainable aquafeed industry. Animals, 2021, 11(3), pp. 1–38, 811

Line 57: Added as reference 5

3. Line 58: Support with Aquaculture, 2020, 522, 735136, Fish and Shellfish Immunology, 2021, 111, pp. 111–118

Line 58 : added both as references 8 and 9 respectively.

4. All scientific names present in the references should be written italic (Please see the attached word file).

We have gone through and made sure they are all italicized. 

Reviewer #2: 

In this paper authors analyzed the profile of microRNAs in all the developmental stages of Hermetia illucens. The aim was to relate them to possible functions in order to understand, in future works, the specific gene regulation to improve mass rearing techniques. I have some doubts that must be addressed, especially regarding the comparison among different life stages, it is not clear, and it seems that in many points sentences seem to be contradictory.

We have made addressed each of the issues raised below and tried to clarify our analyses between life stages. 

1. KEYWORDS: I suggest to change the keywords that are already in the title

Not sure if you are suggesting removing some of the keywords and limit them to just the ones found in our title? 

2. INTRODUCTION: “Black soldier fly larvae (BSFL) contain 42% protein, 35% crude fat (7), and 8.72% chitin in prepupae (8).” This sentence is too generic, protein, lipid and even chitin content change according to the feeding substrates. I suggest to report some examples of different substrates and a range of protein, lipid and chitin content (for example https://doi.org/10.1038/s41598-019-46603-z; https://doi.org/10.1007/s12649-019-00924-2; https://doi.org/10.3390/su131810198)

Authors wrote “they are an ideal candidate for large scale rearing for food sustainability and waste reduction.” Although few studies are performed at industrial scale, I suggest to cite them (for example https://doi.org/10.1038/s41598-020-76571-8; https://doi.org/10.3390/ani10071243)

Fixed Lines 61-68: “The black soldier fly (Hermetia illucens; BSF) is a species found worldwide and can be used as a sustainable animal and fish feed (6-9). Depending on the organic substrate the black soldier fly larvae (BSFL) are fed and the extraction process , they can contain a range of 40-60% protein, up to 47% lipid , and 3-8% chitin in prepupae (10-15). This makes them not only a good protein source, but shows potential for a variety of derived products, and has minimal environmental impacts. Due to these facts, and that they are not a nuisance species, or mechanical disease vector (16, 17), they are an ideal candidate for large scale rearing for food sustainability and waste reduction (18, 19).”

We have added in references suggested, and a range for the composition of prepupae that have been found on different substrates. 

3. RESULTS: In the sentence “A total of 44 miRNAs (23% of the total microRNAs) showed differential expression between at least two life stage with an LFC” write “stages”

Corrected Line 128: “A total of 44 miRNAs (23% of the total microRNAs) showed differential expression between at least two stages with an LFC of ±2.”

4. DISCUSSION: 

What does it mean “Focusing on arthropod species only”? Which species? I don’t understand as the authors said that they performed comparisons with arthropod species.

We changed the sentence at lines 172-173 as follows: “Focusing our miRNA discovery pipeline on known arthropod species miRNA only, allowed for a stronger comparative analysis of potential microRNA function in BSF.” 

“Only three conserved microRNAs were found to be specific to a single life stage: hil-miR-6-2-5p (Egg), hil-miR-3884-3p (Larva), and hil-miR-12418-3p (Pupa).” In the adult stages there are exclusively common microRNAs? Moreover, as the authors identify stage-specific microRNAs, I suggest to perform also a comparison among common microRNAs in all the analysed stages and not exclusively Egg to Larva, Larva to Pupa, Pupa to Female, and Pupa to Male.

We have changed that sentence to be accurate to the conserved miRNA. An error had occurred. 

Line 189-190: “Seventy-four conserved microRNAs were found to be specific to a single life stage, while only 27 were found across all life stages (S1 Table).” 

We are not sure about the second statement on performing a comparison among common miRNAs in all the “analysed stages and not exclusively…” As this is a baseline study showing profile of these novel miRNAs, we did not think a comparison of the miRNAs without a high LFC that were found commonly across all life stages would be significant for publication. We included them and their expression values in the supplemental tables, S3 & S4.

“The unique microRNAs identified had a higher number of life stage specific microRNAs: (Egg) hil-miR-m, hil-miR-p, hil-miR-r, hil-miR-s, hil-miR-u, (Pupa) hil-miR-ac, (Female) hil-miR-h, (Male & Female) hil-miR-a, hil-miR-b, and hil-miR-y.” This sentence is not really clear: did the adult stage have or not specific microRNAs?

We removed “higher” from the text for clarity at line 191. The adult stages had specific unique miRNAs, but the female adult had one unique miRNA not found in the male adult. We could combine them so it just states “adult” but felt this was more specific. 

“However, these 10 unique microRNAs provide insight into the egg, pupa, and adult life stages of the BSF and can be used to compare the developmental regulatory differences of microRNAs to those of other insect species (26)” and what about the larval stage?

Line 196: Added “larva” into the stages. 

“In all three cases the females had expression whereas the males had none.” What does this sentence mean?

Line 201: We removed this sentence. 

“miRNA target prediction” why did the authors choose to explore in detail only 3 microRNAs?

The three explored were selected for their direct implications to mass rearing which we thought had the most impact on the thesis of the paper. 

We added the following at line 220: “the target genes discussed below were selected for having the greatest potential impacts on the mass rearing of BSF”.

This sentence needs a reference “The microRNA has been seen to increase in expression over the third larval instar, with highest expression levels in the pupa stage, in D. melanogaster.”

Line 236: Added reference 44 at the end the sentence.

“Once validated, the microRNA can be harnessed in breeding protocols to improve mass rearing of BSF.” please provide the readers concrete ideas of using these microRNA to improve BSF mass rearing.

Lines 249-252: Once validated, the microRNA can be harnessed in breeding protocols to improve mass rearing of BSF by understanding how they adapt to different environments (28, 46, 54) and is being used in the grape and olive industries (55, 56).”

5. METHODS: Please specify the composition of “standardized proprietary diet”.

Lines 263-264: The flies were fed ad libitum on a standardized proprietary diet based on the commercial composition of layer hen feed. 

Why did the authors choose to perform specifically these comparisons? “Five different paired life stages were compared: Egg to Larva, Larva to Pupa, Pupa to Female, and Pupa to Male.”

Line 282: We have changed the wording in the methods section by removing the word “paired” to show that we did not only compare Egg to Larva, Larva to Pupa, Pupa to Female, and Pupa to Male. In fact, we did all versus all comparison. The Log fold change of all these comparisons for the Unique miRNA (added as supplementary Table 3 and file labelled S3 Table), and the evolutionary conserved miRNA (added as supplementary Table 4 and file labelled S4 Table). 

We have subsequently edited Figure 2 so that it only shows the statistically significant fold changes for the unique miRNAs.

6. FIGURE: “Finally, 21 microRNA were identified with an LFC of ±10 (11% of total miRNA). These included 18 miRNAs conserved across arthropods and 3 miRNAs unique to BSF (Figure 1; Table 3).” In figure 1 only 18 microRNAs were represented. Please fix this, moreover, looking at the image, I supposed a comparison among all the samples. As I wrote before, it is not clear in the main text.

Line 135: We have fixed the figure legend: Figure 1. Heatmap of conserved microRNA expression with a log fold change (LFC) of ±10 (p-adjusted value <0.05) across life stages.

Attachment Submitted filename: ResponseLetter_PONE-D-21-33119.pdf Click here for additional data file.

# Peer review: Decision Letter 1

**DOI:** 10.1371/journal.pone.0265492.r003

**Authors:** Patrizia Falabella

3 Mar 2022

microRNA profile of Hermetia illucens (Black Soldier Fly) and implications on mass rearing

PONE-D-21-33119R1

Dear Dr. Christoffels,

We’re pleased to inform you that your manuscript has been judged scientifically suitable for publication and will be formally accepted for publication once it meets all outstanding technical requirements.

Within one week, you’ll receive an e-mail detailing the required amendments. When these have been addressed, you’ll receive a formal acceptance letter and your manuscript will be scheduled for publication.

An invoice for payment will follow shortly after the formal acceptance. To ensure an efficient process, please log into Editorial Manager at http://www.editorialmanager.com/pone/, click the 'Update My Information' link at the top of the page, and double check that your user information is up-to-date. If you have any billing related questions, please contact our Author Billing department directly at authorbilling@plos.org.

If your institution or institutions have a press office, please notify them about your upcoming paper to help maximize its impact. If they’ll be preparing press materials, please inform our press team as soon as possible -- no later than 48 hours after receiving the formal acceptance. Your manuscript will remain under strict press embargo until 2 pm Eastern Time on the date of publication. For more information, please contact onepress@plos.org.

Kind regards,

Patrizia Falabella

Academic Editor

PLOS ONE

Additional Editor Comments (optional):

Reviewers' comments:

Reviewer's Responses to Questions

**Comments to the Author**

1. If the authors have adequately addressed your comments raised in a previous round of review and you feel that this manuscript is now acceptable for publication, you may indicate that here to bypass the “Comments to the Author” section, enter your conflict of interest statement in the “Confidential to Editor” section, and submit your "Accept" recommendation.

Reviewer #1: All comments have been addressed

Reviewer #2: All comments have been addressed

**********

2. Is the manuscript technically sound, and do the data support the conclusions?

The manuscript must describe a technically sound piece of scientific research with data that supports the conclusions. Experiments must have been conducted rigorously, with appropriate controls, replication, and sample sizes. The conclusions must be drawn appropriately based on the data presented. 

Reviewer #1: Yes

Reviewer #2: Yes

**********

3. Has the statistical analysis been performed appropriately and rigorously? 

Reviewer #1: Yes

Reviewer #2: N/A

**********

4. Have the authors made all data underlying the findings in their manuscript fully available?

The PLOS Data policy requires authors to make all data underlying the findings described in their manuscript fully available without restriction, with rare exception (please refer to the Data Availability Statement in the manuscript PDF file). The data should be provided as part of the manuscript or its supporting information, or deposited to a public repository. For example, in addition to summary statistics, the data points behind means, medians and variance measures should be available. If there are restrictions on publicly sharing data—e.g. participant privacy or use of data from a third party—those must be specified.

Reviewer #1: Yes

Reviewer #2: Yes

**********

5. Is the manuscript presented in an intelligible fashion and written in standard English?

PLOS ONE does not copyedit accepted manuscripts, so the language in submitted articles must be clear, correct, and unambiguous. Any typographical or grammatical errors should be corrected at revision, so please note any specific errors here.

Reviewer #1: Yes

Reviewer #2: Yes

**********

6. Review Comments to the Author

Please use the space provided to explain your answers to the questions above. You may also include additional comments for the author, including concerns about dual publication, research ethics, or publication ethics. (Please upload your review as an attachment if it exceeds 20,000 characters)

Reviewer #1: The authors have properly addressed the comments in the revised manuscript. The manuscript merits acceptance in its present form

Reviewer #2: I thank the authors for improving the manuscript according to my suggestions.

Regarding the keyword, generally they must not be equal to the words of title and this helps to improve the search of the manuscript during a website research. It was just a piece of advice, but it is not fundamental and the manuscript can be accepted in this form.

**********

7. PLOS authors have the option to publish the peer review history of their article (what does this mean?). If published, this will include your full peer review and any attached files.

If you choose “no”, your identity will remain anonymous but your review may still be made public.

**Do you want your identity to be public for this peer review?** For information about this choice, including consent withdrawal, please see our Privacy Policy.

Reviewer #1: No

Reviewer #2: No

# Editor report: Acceptance letter

**DOI:** 10.1371/journal.pone.0265492.r004

**Authors:** Patrizia Falabella

8 Mar 2022

PONE-D-21-33119R1 

microRNA profile of *Hermetia illucens* (black soldier fly) and its implications on mass rearing 

Dear Dr. Christoffels:

I'm pleased to inform you that your manuscript has been deemed suitable for publication in PLOS ONE. Congratulations! Your manuscript is now with our production department. 

If your institution or institutions have a press office, please let them know about your upcoming paper now to help maximize its impact. If they'll be preparing press materials, please inform our press team within the next 48 hours. Your manuscript will remain under strict press embargo until 2 pm Eastern Time on the date of publication. For more information please contact onepress@plos.org.

If we can help with anything else, please email us at plosone@plos.org. 

Thank you for submitting your work to PLOS ONE and supporting open access. 

Kind regards, 

PLOS ONE Editorial Office Staff

on behalf of

Prof. Patrizia Falabella 

Academic Editor

PLOS ONE
